# Universal health coverage for women of reproductive ages: a survey-based comprehensive assessment of service utilisation and health expenditure in Tanzania

**DOI:** 10.1136/bmjph-2023-000672

**Published:** 2025-01-16

**Authors:** Sophia Adam Kagoye, Mark Urassa, Charles Mangya, Coleman Kishamawe, Jim Todd, Milly Marston, Peter Binyaruka, Ties Boerma

**Affiliations:** 1 National Institute for Medical Research Mwanza Research Centre, Mwanza, Mwanza, Tanzania, United Republic of; 2 Epidemiology and Biostatistics, Catholic University of Health and Allied Sciences, Mwanza, Mwanza, Tanzania, United Republic of; 3 Population Health, London School of Hygiene and Tropical Medicine, London, UK; 4 Population Health, London School of Hygiene & Tropical Medicine Department of Population Health, London, UK; 5 Ifakara Health Institute, Dar es Salaam, Tanzania, United Republic of; 6 Global Health, University of Manitoba, Winnipeg, Manitoba, Canada

**Keywords:** Public Health, Community Health, Cross-Sectional Studies

## Abstract

**Introduction:**

Universal health coverage (UHC) for women of reproductive ages is a critical component of country and global health strategies but most evidence in high-fertility settings is limited to maternity care. Our study aimed to comprehensively assess women’s health service utilisation and expenditure, including an equity dimension.

**Methods:**

We conducted a household survey among 15–49 years as a nested study within the Magu health and demographic surveillance study, northwest Tanzania, during 2020–2021. Data were collected on self-reported health, fertility, utilisation of health services, health expenditure and health insurance. We analysed key indicators by household wealth quintiles, place of residence and health insurance, using logistic regression models controlling for age and other confounders.

**Results:**

Among 8665 women aged 15–49 years (response rate 81%), 3.0% reported poor or very poor health, 13% gave birth in the preceding year, and health insurance coverage was 5.1%. Coverage of antenatal (99.5%) and institutional delivery care (88%) were high; 7.3% of women reported at least one outpatient visit in the last 4 weeks, of which 81% were for their own non-maternal healthcare; 9.3% had been admitted to a hospital during the last year, and 74% of these admissions were for deliveries. The total average annual health expenditure per woman was about TZS 16 860 (US$7.50), of which 82% was for her own healthcare and 18% for maternity care. Additionally, women spent about TZS 23 172 (US$10.00) per year on self-treatment. The poorest women had poorer self-reported health, lower coverage of maternity care, lower utilisation of services for their own healthcare and lower health insurance coverage, and limited their expenditure by making greater use of nearby public services than richer women.

**Conclusion:**

Women spent more financial resources on their own non-maternal healthcare than maternity care with poorer women still facing disadvantages for their own healthcare. Health insurance programmes were hardly but were associated with an increase in service use. Comprehensive assessments of women’s health needs, service use and expenditures with an equity focus are crucial for shaping UHC strategies tailored to women of reproductive ages.

**WHAT IS ALREADY KNOWN ON THIS TOPIC**

- Universal health coverage (UHC) strategies require comprehensive data on service utilisation and expenditure. Most research on women in high fertility countries is preoccupied with reproductive health. Our analysis aimed to investigate how to go beyond maternal healthcare and simultaneously consider women’s own healthcare issues, while focusing on inequalities by wealth and other dimensions, using survey data.

**WHAT THIS STUDY ADDS**

- Our study in northwest Tanzania underscores the utility of incorporating a module on health service utilisation and expenditure within household surveys, which provide invaluable insights for shaping UHC strategies tailored to women of reproductive ages. Most health expenditure was directed towards outpatient services for the women’s own health needs, rather than maternal healthcare or hospital admissions. Health insurance coverage remained limited. The analysis highlights substantial inequalities across all indicators. Economically disadvantaged women employed various short-term strategies to manage expenditures such as refrain from seeking services, relying on nearby facilities, avoiding private and urban facilities, use of self-medication and foregoing community health insurance.

**HOW THIS STUDY MIGHT AFFECT RESEARCH, PRACTICE OR POLICY**

- Household survey modules are useful instruments to inform efforts to reach all women with adequate and affordable services that address both maternal and own health needs in the context of UHC. Programmes need to provide a more holistic approach to health of women of reproductive ages, with a focus on disadvantaged women whose households cannot afford the costs of health insurance.

## Introduction

 Universal health coverage (UHC), defined as all people receiving quality essential services without incurring financial hardship,^1^ has gained significant attention in global health policy. Many countries, including those in sub-Saharan Africa, are prioritising comprehensive approaches to ensure reach to all citizens.^2^

In high fertility settings, as in most of sub-Saharan Africa, maternity-related care is a major reason for seeking health services among women of reproductive age. Much is known about coverage of maternity services such as antenatal and childbirth-related services through national household surveys.^3 4^ Multiple studies have assessed out-of-pocket expenses associated with maternity care, even in settings with no direct service fees for antenatal care or deliveries.^5^,^7^ Other research has focused on prepayment mechanisms such as health insurance to enhance access to maternity care and reduce out-of-pocket payments.^8^ Yet, health insurance coverage among women of reproductive ages has been limited in most countries in sub-Saharan Africa, and is even lower among women of lower socioeconomic status and rural women.^9 10^ To date, few studies have offered a more holistic picture of women’s healthcare needs, service utilisation and health expenditure, encompassing both maternity care and overall women’s health not related to maternity care.

In Tanzania, the 5-year national Health Sector Strategic Plan V (HSSP V) aims to make substantial progress towards UHC.^11^ This requires efforts towards high and equitable levels of coverage with essential health services and protection from catastrophic out-of-pocket health expenses.^12 13^ The 2022 Tanzania Demographic and Health Survey (TDHS) showed that coverage of maternity care was high in Tanzania with 65% of pregnant women making at least four antenatal care visits and 81% of births in health facilities in the preceding 2 years.^14^ The 2016 TDHS reported an average annual healthcare expenditure per woman, aged 15–49 years, of TZS 11 442 (US$4.97), and a health insurance coverage of 9% among women of reproductive ages, including 5% through community-based health insurance, which has been a key strategy for financial protection and service utilisation in Tanzania.^15 16^

In this study, we aimed to examine the extent to which a local household survey module could provide insights into women’s health service coverage and utilisation, health expenditure and insurance coverage to inform UHC strategies. Our assessment considered both maternity care and women’s own non-maternity-related healthcare, as well as inequalities in coverage and financing and strategies employed by women to manage healthcare expenses.

## Methods

### Study setting

**Figure 1:**
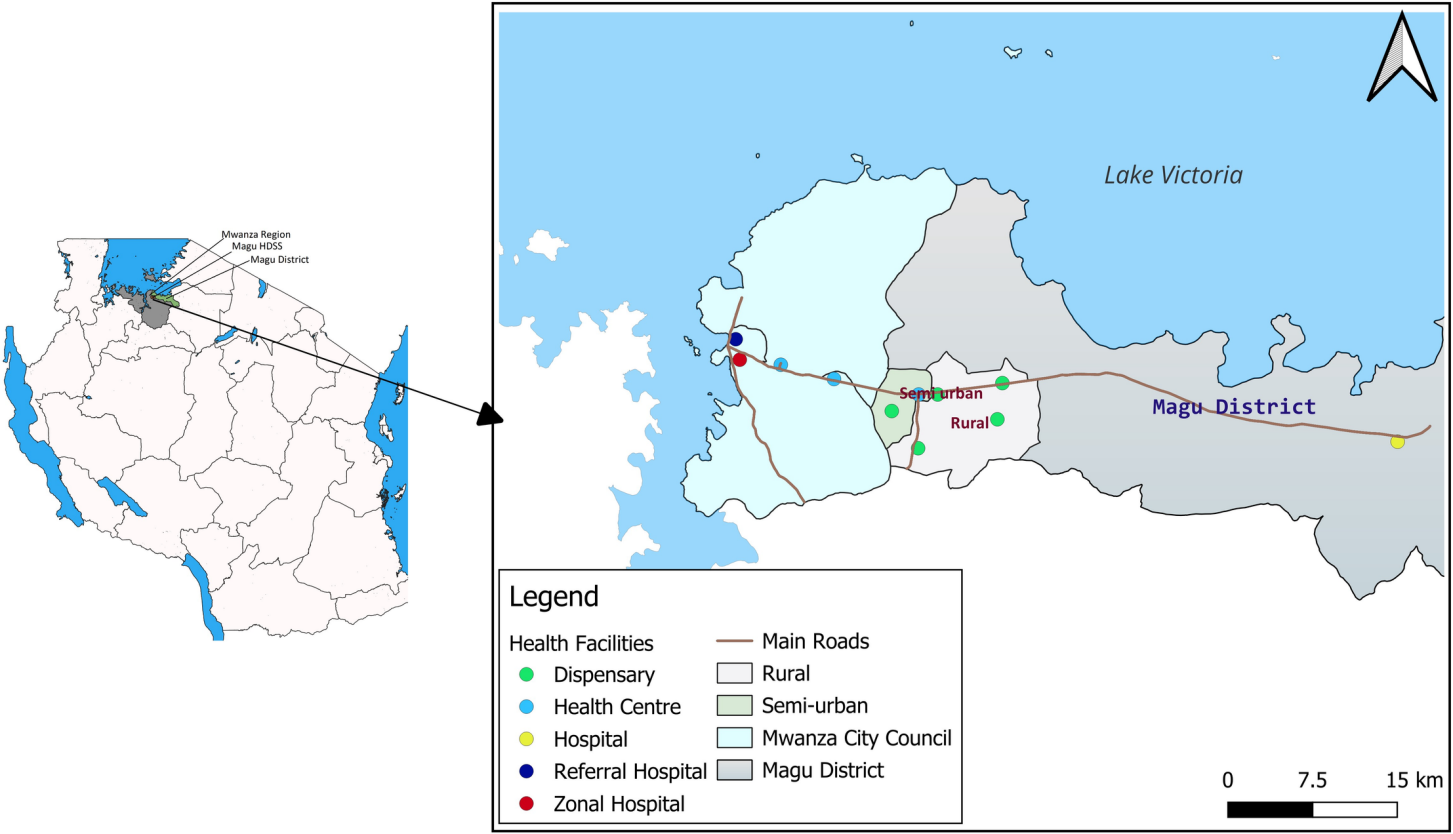
Map of Magu HDSS showing semiurban and rural boundaries and the distribution of health facilities. HDSS, health and demographic surveillance system

The study was conducted as a household survey nested within the Magu health and demographic surveillance system (HDSS), which has been operational and running since 1994 in a contiguous area of nine villages (four semi-urban and five rural) in northwest Tanzania, 20 km east of the city of Mwanza, with a population of 50 254 in 2021.^17^ The demographic surveillance rounds are conducted on average at 8-month intervals. The Magu HDSS area has six government health facilities, including one health centre in the main roadside trading centre and five dispensaries in smaller villages, and one private clinic. All six government facilities provide antenatal and delivery care services. The regional hospital, zonal referral hospital and multiple private health facilities are located in Mwanza city (figure 1). Further details of the Magu HDSS demographic and socioeconomic characteristics with regional and national comparisons are described elsewhere.^17 18^

### Data source

We conducted a household survey nested within the Magu HDSS among all women of reproductive ages (15–49 years) from October 2020 to November 2021. The listing of eligible women was extracted from the 37th round of the demographic surveillance system in early 2020. The field work was interrupted for a period of 4 months (from April 2021 to July 2021) due to travel restrictions associated with the COVID-19 pandemic. All eligible women 15–49 years were interviewed in their own household. The survey instrument focused on reproductive, maternal, newborn and child health, and was a short version with several adaptations from the selected modules of Demographic and Health Surveys Women questionnaire.^19^

The survey interview lasted, on average, about 45 min and included modules on background household and individual characteristics, birth history, outpatient and inpatient service utilisation and expenditure (woman), family planning, antenatal and delivery care, healthcare for children born in the 3 years prior the survey. The survey instrument is included as a online supplemental material. Most modules were adapted version of the standard Demographic and Health Surveys (DHS) Questionnaire. Differences included the addition of questions in the birth history about health seeking behaviour for all sick children under 20 years of age, direct administration of the household schedule and utilisation and expenditure modules to the female respondent rather than the head of household as is the case in the DHS, the use of specific codes for named local health facilities, and the focus on the last event with a reference period rather than all events (to reduce interview length and complexity) (online supplemental table S1).

The survey instrument was administered using an electronic tablet, and included five modules: demographic and socioeconomic background, full birth history, service utilisation and expenditure for the respondent’s own health, coverage of reproductive, maternal and child health services and COVID-19 pandemic’s knowledge, attitude and response. The interviewers were trained for 2 weeks and conducted a pilot to improve their skills. Data processing was done concurrently with data collection to allow for the generation of weekly field data quality checks. The tables were discussed with the field supervisor who then followed up with the interviewers.

This study focused on the following dimensions of the women’s health:

1. General healthcare needs: self-reported health on the day of interview, using a five-point ordinal scale (from very poor to very good), and fertility based on the birth history data. We computed both total fertility rate (number of children per woman) and the general fertility rate, defined as the annual number of births per 1000 women 15–49 years for the year prior the survey.
2. Coverage of maternity services: antenatal care visits and health facility delivery for live births in the last 3 years, current use of modern contraceptives and cervical cancer screening in the last 3 years for women 30 years and older. The term coverage of services/care used in this study refers to contact coverage, defined as the ratio of the number receiving the intervention by those who need it.
3. Health service utilisation: outpatient department (OPD) visits in the last 4 weeks and inpatient admissions in the last 12 months, by health facility type and location, and general reason for seeking care, grouped into maternity care (including family planning) and own healthcare (disease, injury).
4. Health expenditure: expenses associated with most recent OPD visit and admissions within the reference period. The respondent’s expenditure for self-treatment outside the formal health system in the last 4 weeks was also considered.
5. Health insurance: the respondent’s or household insurance coverage from any type of insurance scheme in Tanzania—National Health Insurance Fund (NHIF), Community Health Fund (CHF), National Social Security Fund (NSSF) and employer-based or other private insurance.

Detailed description of indicators analysed in this study is outlined in online supplemental table S2.

### Dimensions of inequality

To assess inequalities in service coverage and health expenditure, we stratified the analyses by residence, household wealth and health insurance coverage. The women residing in the trading centre villages along the main road (and nearer to the city) were classified as semiurban (49% of the study population), while the remaining 51% were considered rural. Household wealth was estimated using a principal component analysis with a list of household assets and characteristics^20^ derived from a module implemented in demographic surveillance round 33 in 2017. The linking of household wealth data with the survey was successful for 80% of respondents, which were then classified in wealth quintiles.

### Data analysis

Data were cleaned using the Census and Survey Processing System software (CS-Pro) and analysed with Stata V.18.^21^ We used χ^2^ to compare indicators of health needs and service utilisation by the selected determinants. Factors associated with health service utilisation (OPD utilisation and admissions for maternity or own healthcare) were assessed using binary logistic regression models, summarised with adjusted odds ratios (AORs) and corresponding 95% CI. These models included individual (eg, women’s age) and household variables (eg, wealth). We did not correct for intrahousehold clustering effects.

The expenditure for OPD utilisation was calculated from the mean reported costs for the last visit (including those with zero costs) and the annual number of OPD visits (by multiplying the number of visits in the last 4 weeks by 13). The annual expenditure on admissions was derived from the total number of admissions per woman in the last year and mean expenditure based on the last admission. The total per capita annual health expenditure was computed as the sum of the per capita annual costs associated with OPD visits and inpatient service use. All expenditure data were reported in the local currency, Tanzanian shilling (TZS) (1 USD=2300 TZS, average exchange rate during 2020–2021).

In all analyses p values <0.05 were considered statistically significant. Goodness of fit of the models was checked using Akaike information criteria (AIC), considering a lower AIC value. All findings were presented in respective tables and figures.

### Patient and public involvement

There was no patient or public involvement in the design, implementation or reporting of this study.

## Results

In total, 8665 women 15–49 years were interviewed. The response rate was 81%, with little variation by background characteristics. The median age was 27 years, 57% were currently married and 82% had at least primary education, including 35% with secondary education or higher (online supplemental tables S3 and S4).

### General health and coverage

Self-reported health was poor or very poor for 3.0% of women, and more common among rural women and the poorest women compared with semiurban and richer women, respectively (table 1). Poor self-reported health with functioning limitations was reported by 1.5% of women, with further widening of the inequalities. About 13% of women gave birth in the year before the survey, ranging from 10% to 16% among the women in the richest and poorest wealth quintiles, respectively. Fertility differed by age with a peak in women aged 15–34 years. The total fertility rate was 4.6 children per women for the 3 years preceding the survey.

**Table 1:** General health status, coverage of selected indicators by age of the respondent, household wealth quintile, place of residence and health insurance status among women 15–49 years in Magu Health and Demographic System, 2020–2021

General health and fertility indicators					
	N	General health	Fertility		
		Very poor/poor self-reported health	With functional limitations	Gave birth last year	Total fertility rate
Total number of women	8665	3	1.5	13.5	4.6
Age					
15–24	3586	1.8	0.9	12.5	
25–34	2482	3.0***	1.3***	20.4	
35	2597	4.7***	2.7***	8.3	
Area of residence					
Semiurban	4091	2.1	1	11.7	3.7
Rural	4574	3.9***	2.1***	15.1	5.5
Wealth					
Poorest	1494	4.6***	2.6***	15.6	6.1
Richest	1370	2.5	1.1	9.6	3.1
Insurance					
No	8216	3	1.6	13.5	4.6
Yes	440	3.4	1.6	13.2	4.8

Service coverage						
	N	ANC: at least one visit	ANC: at least four visits	Health facility births	Modern contraceptive use	Cervical cancer screening
Total number of women	8665	99.5	72.1	87.9	23	10.9
Age						
15–24	3586	99.5	72.9	90.9	19.6	–
25–34	2482	99.5	74	85.8***	27.4***	9.3
35	2597	99.8	61	77.9***	20.6***	11.4
Area of residence						
Semiurban	4 091	99.6	74.3	93.5	25.5	13.7
Rural	4574	99.3	70.6	84.2***	21.0***	8.0 ***
Wealth						
Poorest	1494	99.3	74.4	84.3***	15.4***	5.2***
Richest	1370	100	72.7	96.7	27.9	14.7
Insurance						
No	8216	99.5	71.2***	87.7*	22.8	10.2***
Yes	440	100	93.3	93.6	25.7	22.2

OPD service use in the last 4 weeks				
	N	Total	Maternity care	Own healthcare
Total number of women	8665	7.3	1.4	5.9
Age				
15–24	3586	6.7	1.5	5.2
25–34	2482	8.4*	2.1***	6.3
35	2597	7.1*	0.6***	6.5

OPD service use in the last 4 weeks				
Stratifiers	N	Total	Maternity care	Own healthcare
Area of residence				
Semiurban	4091	6.1	0.8	5.3
Rural	4574	8.3***	1.9***	6.4*
Wealth				
Poorest	1494	8	1.8	6.2
Richest	1370	7.4	0.7	6.7
Insurance				
No	8216	6.9***	1.3*	5.6***
Yes	440	15	2.5	12.5

Admitted to health facility in the last year				
	N	Total	Maternity care	Own healthcare
Total number of women	8665	9.3	6.9	2.4
Age				
15–24	3586	8.7	6.9	1.8
25–34	2482	13.0***	10.3***	2.7
35	2597	6.6*	3.7***	2.9
Area of residence				
Semiurban	4091	9.3	6.6	2.7
Rural	4574	9.3	7.2	2.1
Wealth				
Poorest	1494	8.1	6.6	1.5
Richest	1370	8.2	6.1	2.1
Insurance				
No	8216	9.2	6.9	2.3
Yes	440	11.1	7.7	3.4

Health insurance coverage			
	N	Any type of insurance	Community health fund
Total number of women	8665	5.1	1.3
Age			
15–24	3586	4.9	1.8
25–34	2482	4.9	0.9***
35	2597	5.6	1.1***
Area of residence			
Semiurban	4091	4091	1.2
Rural	4574	4574	1.5***
Wealth			
Poorest	1494	2.5***	1.5***
Richest	1370	9.2	0.9

χ2 p values: *p<0.05; **p<0.01; ***p<0.001.

Total fertility rate is adjusted by age groups.

OPD, outpatient department.

Antenatal and delivery care coverage were high with nearly 100% for ANC first visit, 72% for at least four ANC visits and 88% of births in health facilities (table 1). Modern contraceptive use among currently married women was 23%. Coverage of cervical cancer screening in the last 3 years among women 30–49 years was low (11%). Facility births were lower among the poorest women (84%) compared with 97% among the richest women. Also, modern contraceptive use and cervical cancer screening were lower among the poorest women compared with women from wealthier households. However, there were few inequalities for antenatal care utilisation (table 1).

Health insurance coverage was 5% among women aged 15–49 years, ranging from 2.5% among the poorest women to 9% among the richest. The community health fund coverage was below 2% for all categories (table 1).

### Outpatient and inpatient utilisation

Overall, 7.3% of women reported at least one OPD visit to a health facility in the last 4 weeks, of which 81% of the visits were for their own health and 19% for maternity care. The mean number of visits per woman per year was 1.4, including revisits. About 1 (9.3%) in 11 women had been admitted to a hospital during the last year, and 74% of these admissions were for maternal healthcare and 26% for her own healthcare (table 1).

**Figure 2:**
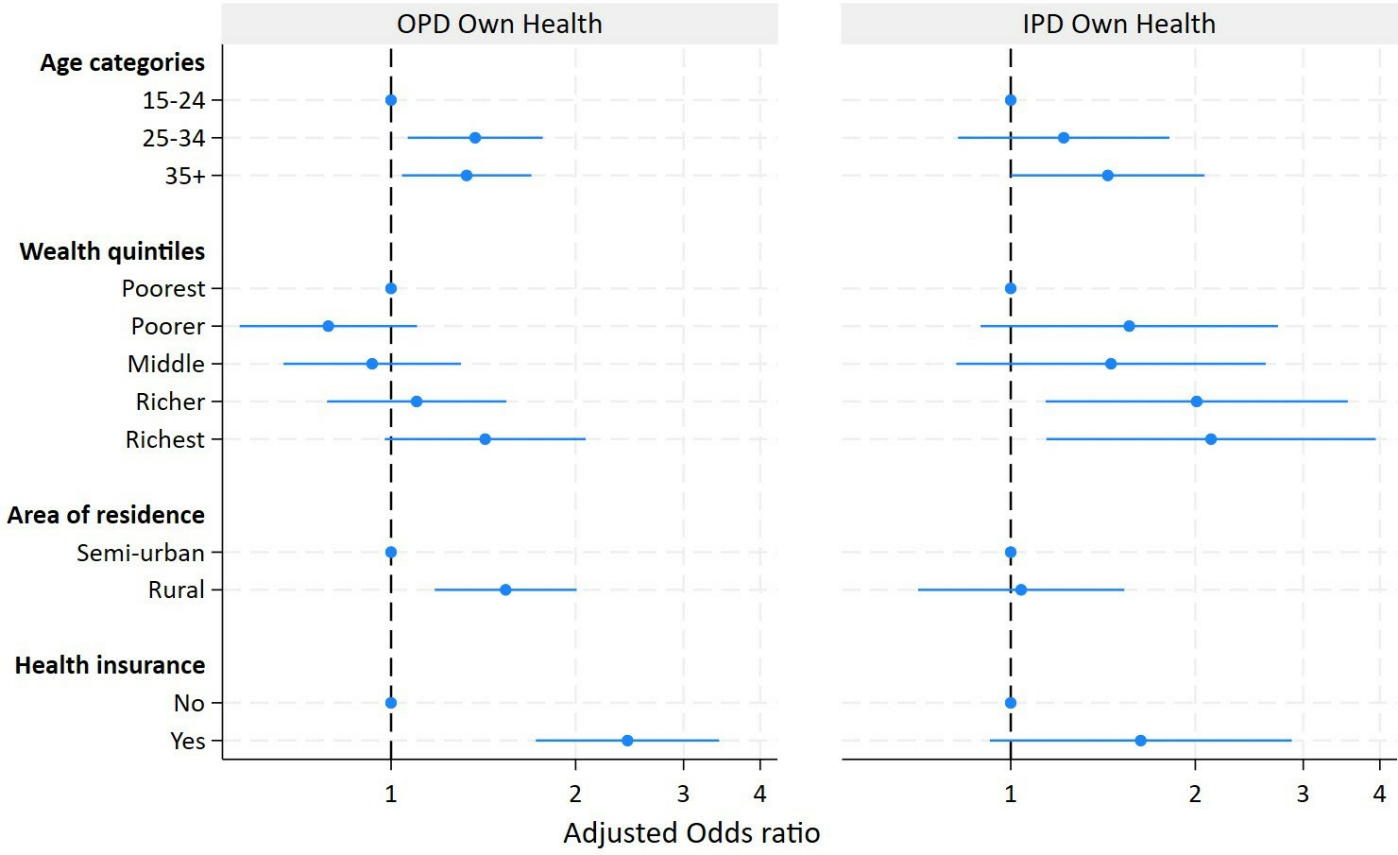
Adjusted ORs of using outpatient and inpatient service utilisation for own non-maternal health issues by age of the respondents, household wealth quintile, place of residence and health insurance status among women 15–49 years in the Magu Health and Demographic System, 2020–2021 (full model). IPD, inpatient department; OPD, outpatient department.

OPD visits for their own health was higher among women with health insurance (adjusted odds ratio (AOR): 2.43; 95% CI: 1.72, 3.43), rural women (AOR: 1.54; 95% CI: 1.18, 2.01) and among the richest women, though not statistically significantly (AOR: 1.42; 95% CI: 0.98, 2.08) (figure 2). Admissions for the woman’s own health were significantly more common among women in the fourth (AOR: 2.01; 95% CI: 1.14, 3.55) and fifth wealth quintiles (AOR: 2.12, 95% CI: 1.14, 3.94) compared with women in the poorest quintile. Women with health insurance were more frequently admitted for their own healthcare than women without insurance (AOR: 1.63; 95% CI: 0.92, 2.87), although not statistically significant and no differences were observed by place of residence (figure 2). Online supplemental tables S5 and S6 present the OR for the different models for both outpatient and admissions for maternity and own health, respectively).

### Location of services

For OPD services, women primarily used health facilities within the study area (63% of OPD visits), with the remainder using private facilities in or outside the area (28%) or Mwanza city public facilities (7%). For admissions, more than 60% used the local health centre, 28% the large hospitals in Mwanza city and 8% private facilities (online supplemental figure S1). Women in the richest wealth quintile and those residing in semiurban areas had much higher use of city and private hospitals, while poorer women relied more on the local government health centre for OPD services. Semiurban, richer and insured women had greater use of public hospitals and private facilities in the city for admissions, while rural residence and lower wealth quintile were admitted to the health centre and sometimes dispensaries in the study area.

**Figure 3:**
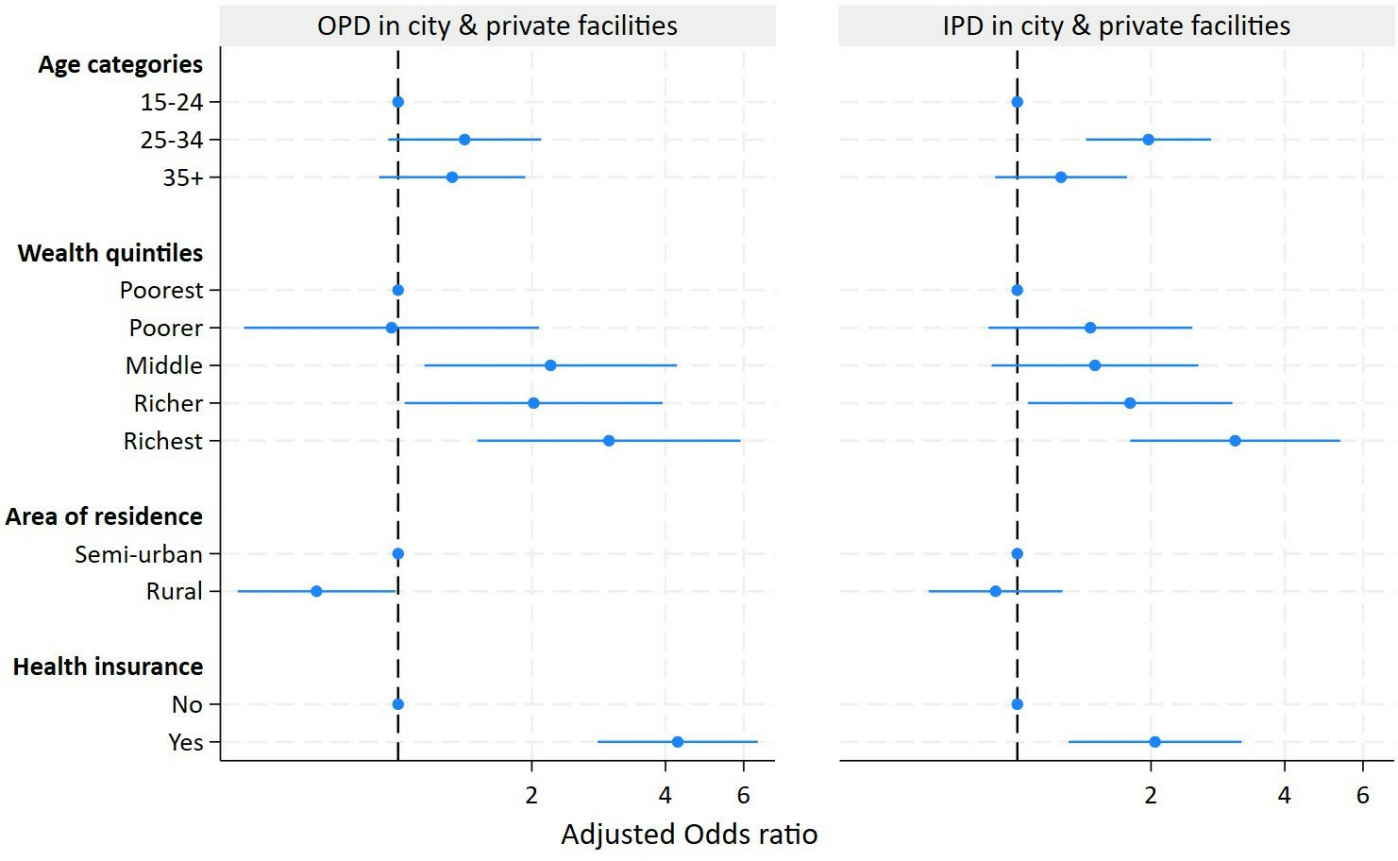
Adjusted OR of using outpatient and inpatient health facilities in Mwanza city. Per cent distribution of type of facility for outpatient (top panel) and inpatient (bottom panel) service utilisation, by household wealth quintile, place of residence and health insurance status, among women 15–49 years, Magu Health and Demographic Surveillance Study, 2020–21 (full model). OPD, outpatient department, IPD, inpatient department

The type and location of the facilities for admission differed markedly by background characteristics. Insured women (AOR: 2.04; 95% CI: 1.30, 3.20), women from the richest quintile (AOR: 3.09; 95% CI: 1.79, 5.33) and those aged 25–34 years (AOR: 1.97; 95% CI: 1.43, 2.73) were more likely to attend Mwanza city and private facilities. For OPD services, private facilities were also more frequently used by the richest women (AOR: 2.98; 95% CI: 1.51, 5.91), semiurban women and women with health insurance (AOR: 4.26; 95% CI: 2.81, 6.45) (figure 3, online supplemental table S7).

### Health expenditure

The total average annual expenditure on health services per woman was TZS 16 860. OPD services for her own healthcare accounted for 71% of total annual health expenditure, followed by inpatient maternity care (11%), inpatient care for her own health (11%) and OPD maternity care (7%) (table 2). In total, 77% was for OPD services and 23% for inpatient care, and most expenditure (82%) was related to the woman’s own health service use.

**Table 2:** Total average annual expenditure on outpatient and inpatient service utilisation for maternity or own healthcare in Tanzania Shilling (TZS) with per cent distribution (in parenthesis) by household wealth quintile, place of residence and health insurance status among women 15–49 years in Magu Health and Demographic System, 2020–2021

	OPD maternity care	OPD own healthcare	IPD maternity care	IPD own healthcare	Total
Total	1145 (6.8)	11 904 (70.6)	1947 (11.5)	1864 (11.1)	16 860 (100)
Age					
15–24	1589 (13.4)	7924 (66.8)	1405 (11.8)	941 (7.9)	11 859 (100)
25–34	578 (3.0)	13 124 (68.2)	3591 (18.6)	1962 (10.2)	19 255 (100)
35+	1051 (4.8)	16 421 (75.8)	1107 (5.1)	3098 (14.3)	21 677 (100)
Residence					
Semiurban	1582 (8.3)	13 020 (68.0)	1807 (9.4)	2738 (14.3)	19 147 (100)
Rural	736 (5.0)	10 808 (73.5)	2072 (14.1)	1086 (7.4)	14 702 (100)
Wealth					
Poorest	889 (7.5)	8539 (72.2)	1726 (14.6)	669 (5.7)	11 823 (100)
Richest	2104 (8.6)	16 348 (66.7)	2444 (10.0)	3614 (14.7)	24 510 (100)
Insurance					
No	905 (5.5)	11 914 (72.0)	1811 (10.9)	1918 (11.6)	16 548 (100)
Yes	5909 (26.3)	11 212 (49.9)	4409 (19.6)	960 (4.3)	22 490 (100)

IPD, inpatient department; OPD, outpatient department.

The amount spent on health services increased with age, driven by increased spending on services for the woman’s own healthcare. Large differences were observed by wealth quintile where the expenditure on services was 2.1 times higher among the richest compared with the poorest, spending substantially more on outpatient and inpatient services for both maternity and own healthcare. Women with health insurance spent 1.4 times more on health services than women without insurance. This was due to much higher expenditure on maternity care services (6.5 and 2.4 times higher for OPD services and admission, respectively), while expenditure on admissions for her own health was lower among those with health insurance. The higher per capita expenditure associated with use of maternity inpatient services occurred despite lower fertility and was due to much higher use of health facilities in Mwanza city rather than local public facilities. Women who used delivery services within the Magu HDSS area spent on average TZS 7735, including 57% who reported had no costs at all, compared with TZS 60 665 among those who used delivery services elsewhere.

More than one in five respondents (22%) indicated that they had spent money on buying medicines or other treatments outside of the formal health services in the last 4 weeks. This corresponded with an average annual expenditure of TZS 23 172 per woman for self-treatment, 1.4 times higher than the total formal healthcare services expenditure (online supplemental table S8). Women with health insurance spent 56% of all health expenditure on self-treatment, compared with 58% among those with no insurance. The poorest women spent relatively more on self-treatment (58% and 52% of all health expenditure among the poorest and richest women, respectively).

## Discussion

UHC requires comprehensive data on health needs, service utilisation and expenditure. Our analysis of survey data shows the importance of considering both subjects in a survey that goes beyond maternal healthcare and consider women’s own healthcare issues, as well as focus on inequalities by wealth and other dimensions to inform UHC strategies.

The general picture of our study population shows high fertility, high coverage of maternity care and low health insurance coverage, which is not different from other studies’ findings within Tanzania mainland.^22 23^ Most women (97%) reported average to good health and frequent childbearing with a total fertility of 4.6, slightly below Tanzania’s average of 4.8 in the TDHS 2022. Coverage of health facility births was over 80% and similar to a 2022 national survey.^14^ Health insurance coverage in Magu HDSS was 5%, which was lower than 9% national insurance overage reported in the.^15^ Total annual health expenditure of TZS 16 860 was almost 1.5 times higher than in the TDHS 2016 for the country.^15^

Women used OPD services primarily for her own healthcare (81% of visits), but inpatient admissions were predominantly associated with maternity care (74% of admissions). Just 18% of the per capita total annual expenditure on health services was due to maternity care use. This suggests that Tanzania’s health policy of free basic antenatal and delivery care services in public health facilities^11^ has been successful in keeping the population-level expenditure of the use of maternity care services low, even in the context of high fertility and high levels of maternity care coverage. Achieving UHC for women of reproductive age, however, still needs a stronger enforcement of a fee exemption policy and/or improving health insurance to this population subgroup.

Our findings revealed potential mechanisms that women used to keep health spending low. First, most women used public services that were nearby, meaning dispensaries and health centres within the neighbouring villages. This also saves on transport and time which can be significant expenditures.^23^ Those that used services in Mwanza city had much higher expenses, especially for maternity care. Second, many women relied on self-treatment. Women spent more on self-treatment than on all formal services combined, presumably because it is perceived to be more economical and equally or more effective. In the 4 weeks preceding the survey, 22% of women used self-treatment, while only 7% went to OPD.

Health insurance was not a common strategy to reduce health spending and protect women from potential higher expenses. Small numbers precluded an assessment by type of insurance. Those with any type of health insurance spent 36% more on healthcare services than women without insurance, mainly because they used more city and more private services. Only few women benefitted from health insurance schemes for formal sector employees, such as the NHIF covering civil servants.^16^ The voluntary insurance scheme, the CHF, primarily for rural households offering public primary care to the informal sector, has not become a major instrument for financing of health services and protection of households against health expenses. Insurance coverage was more common among wealthier community members and still associated with higher levels of health expenditure.^10^ Further reasons for low insurance uptake were not investigated in this study but other studies in Tanzania and elsewhere have reported similar findings.^8 22^ Affordability of premiums may be considered a significant financial barrier in a context where income and annual health expenditures are low. The perception of limited perceived short-term benefits may be another contributing factor, as the costs of an annual CHF insurance is TZS 30 000 to a household, not all services are covered by the insurance, and women’s annual spending on health tends to be low.^22^

The socioeconomic and geographic inequalities were substantial for most components of women’s health, including service utilisation, expenditure and insurance coverage, especially for poorer and to a lesser extent rural women compared with richer and semiurban women within the small study area. However, the inequalities for maternity care were much smaller than for own healthcare, which may be taken as further evidence of the success of Tanzania’s maternity care policies.

Our study has several limitations. We did not conduct a full assessment of out-of-pocket expenditures by including all types of costs such as transport costs, loss of income and so on, which is often substantive.^6 7^ Second, due to data limitation on non-health household expenditures and income, we were unable to compute the incidence of catastrophic and impoverishing health spending. The relatively large amount spent on self-treatment requires further investigation as it was based on a single question in our module and needs further investigation. Finally, no detailed assessment of health needs and the quality of services was conducted which requires more detailed survey data (including biomarkers) and health facility assessments.

## Conclusion

Our study shows that a survey module on health service utilisation and expenditure, such as the current modules in the Demographic and Health Surveys, can provide relevant information beyond maternal care that is relevant to UHC strategies for women of reproductive ages. Regular implementation of such modules informs efforts to reach all women with adequate and affordable services that address both maternity and her own health needs.

## Supplementary material

10.1136/bmjph-2023-000672 online supplemental file 1

10.1136/bmjph-2023-000672 online supplemental file 4

## Data Availability

Data are available upon reasonable request.
