# Supplementary material for: Universal health coverage for women of reproductive ages: a survey-based comprehensive assessment of service utilisation and health expenditure in Tanzania
Source: BMJ Public Health. 2025 Jan 16;3(1):e000672. doi: 10.1136/bmjph-2023-000672 (PMC11812853; doi:10.1136/bmjph-2023-000672)
Supplement: online supplemental file 1 [file bmjph-3-1-s001.pdf]

Supplement materials

Table S1: Differences between Demographic and Health Survey Questionnaire and Women Survey questionnaire for key modules used in the study (Service utilization and health expenditure)

**Indicator**	**Demographic and Health Survey Questionnaire**	**Women survey Questionnaire**
Respondent	Proxy reporting by the head of the household	Women themselves (Aged 15-49 years)
Reference period inpatient care	6 months	12 months
Inpatient care (IPD) and Outpatient care (OPD) questions	Number of admissions/outpatient visits within the reference period<br><br>Follow up questions on place of service, reason for visit, expenditure- are asked for all visits within the reference period	Number of admissions/outpatient visits within reference period<br><br>Follow up questions on place of service, reason for visit, expenditure- are asked for the final visit<br><br>Additional questions on timing of the last service utilization and satisfaction with care<br><br>Created specific categories for place of service (health facilities) specific to the study area

Table S2: Indicator definitions

Indicator	*Definition*	*Denominator*
	General health care needs	
Very poor/poor self-reported health	Number of women 15-49 years with very poor/poor self-reported	Total women 15-49 years
Proportion gave birth last year (General fertility rate)	Average number of children currently being born to women 15-49 years in 1 year preceding the survey	Women 15-49 years per 1000 (one year prior the survey)
Total fertility rate	Number of children per woman (calculated for 3 years preceding the surveys)	Women 15-49 years per 1000 (three years prior the survey)
	Coverage of reproductive and maternal health services	
ANC: 1st visit	Percentage of women age 15-49 attended by any provider at least once during pregnancy (ANC1), 3 years prior the survey	Total number of women aged 15−49 years with a live birth in the same period
ANC: at least 4 visits	Percentage of women age 15-49 attended by any provider at least four times during pregnancy (ANC4) , 3 years prior the survey	Total number of women aged 15−49 years with a live birth in the same period
Health facility delivery	Percentage of births in the health facilities, 3 years prior the survey	Total number of women aged 15−49 years with a live birth in the same period
Modern contraceptive use	The percentage of all women 15-49 years who are using (or report their partner is using) a modern contraceptive method in a specific year at a particular point in time.<br>Modern contraceptives methods included: (Female sterilization, male sterilization, Intrauterine Device (IUD), injectable, implants, pill, emergency contraception, male condom, female condom, other vaginal methods (foam, jellies/spermicide, diaphragm), Standard Days Method (SDM), Lactational Amenorrhea Method (LAM))	Currently married women
Cervical cancer screening	Proportion of women 30+ who have been examined for cervical cancer by a doctor/nurse	Total number of women (30+ years)
	Curative care service utilization	
Outpatient (OPD) service utilization	Number of OPD visits in the last 4 weeks prior the survey	Total number women 15-49 years
Inpatient (IPD) service utilization	Number of admissions in the last 12 months prior the survey	Total number women 15-49 years
Health insurance coverage	Insurance coverage from any type of insurance, including: National Health Insurance Fund (NHIF), Community Health Fund (CHF), National Social Security Fund (NSSF), employer based and other private insurance	Total number women 15-49 years

Table S3: Response rate disaggregated by villages and area of residence

	Number of women seen	Number of women censused in HDSS 35	Response rate
**Villages**			
Bujora	881	1,120	78.7
Kisesa	991	1,195	82.9
Wita	386	489	78.7
Igekemaja	636	786	80.9
Kitumba	1,144	1,476	77.5
Kanyama	1,833	2,393	76.6
Isangijo	1,037	1,163	89.2
Ihayabuyaga	963	1,178	81.7
Welamasonga	794	901	88.1
**Area of residence**			
Semi-urban	4,091	5,197	78.7
Rural	4,574	5,504	83.1
**Total**	**8,665**	**10,701**	**81.0**

Table S4: Background Characteristics of study participants (N=8,665)

Characteristic	Frequency	Percentage
**Age**		
15-24	3,586	41.4
25-34	2,482	28.6
35+	2,597	30.0
Median (IQR) 27 (20,37)		
**Area of residence**		
Semi-urban	4,091	47.2
Rural	4,574	52.8
**Marital status**		
Never married	2,843	32.8
Married/Cohabiting	4,944	57.1
Separated/Divorced/Widowed	878	10.1
**Education level**		
None/Incomplete primary education	1,572	18.1
Complete primary education	4,060	46.9
Secondary education and above	3,033	35.0
**Household wealth index (n=6,894)**		
Poorest	1,494	21.7
Poorer	1,310	19.0
Middle	1,315	19.1
Richer	1,405	20.4
Richest	1,370	19.9

Table S5: Factors associated with outpatient service utilization for maternal/own health (Crude & Adjusted Analysis) among women of reproductive age (15-49 years) in Magu Health and Demographic Surveillance site, 2020-2021 (N=8,665)

Characteristic	OPD Maternity		OPD Own health	
	Crude Analysis COR (95% CI)	Adjusted Analysis¥ AOR (95% CI)	Crude Analysis COR (95% CI)	Adjusted Analysis¥ AOR (95% CI)
**Age categories**				
15-24	1	1	1	1
25-34	1.45 (0.99, 2.14)	1.45 (0.92, 2.29)	1.23 (0.99, 1.53)	1.37 (1.06, 1.77) *
35+	0.39 (0.22, 0.70) **	0.47 (0.25, 0.86) *	1.26 (1.01, 1.56) *	1.33 (1.04, 1.69) *
**Wealth index**				
Poorest	1	1	1	1
Poorer	0.71 (0.39, 1.32)	0.76 (0.41, 1.40)	0.76 (0.54, 1.05)	0.79 (0.57, 1.10)
Middle	0.92 (0.52, 1.63)	1.07 (0.60, 1.93)	0.83 (0.60, 1.15)	0.93 (0.68, 1.30)
Richer	0.63 (0.34, 1.17)	0.78 (0.40, 1.52)	0.93 (0.68, 1.27)	1.10 (0.79, 1.54)
Richest	0.36 (0.17, 0.77) **	0.55 (0.22, 1.32)	1.10 (0.81, 1.48)	1.42 (0.98, 2.08)
**Area of residence**				
Semi-urban	1	1	1	1
Rural	2.26 (1.51, 3.37) ***	1.70 (0.98, 2.95)	1.22 (1.02, 1.46) *	1.54 (1.18, 2.01) **
**Health insurance**				
No	1	1	1	1
Yes	1.93 (1.03, 3.61) *	1.34 (0.54, 3.36)	2.42 (1.80, 3.27) ***	2.43 (1.72, 3.43) ***

¥Adjusted for age categories, wealth index, area of residence and health insurance status; **p<0.05; **p<0.01; ***p<0.001*

Table S6: Factors associated with inpatient service utilization for maternal/own health (Crude & Adjusted Analysis) among women of reproductive age (15-49 years) in Magu Health and Demographic Surveillance site, 2020-2021 (N=8,665)

Characteristic	IPD Maternity		IPD Own health	
	Crude analysis COR (95% CI)	Adjusted analysis¥ AOR (95% CI)	Crude analysis COR (95% CI)	Adjusted analysis¥ AOR (95% CI)
**Age categories**				
15-24	1	1	1	1
25-34	1.55 (1.29, 1.86) ***	1.63 (1.32, 2.02) ***	1.46 (1.03, 2.06) *	1.22 (0.82, 1.82)
35+	0.52 (0.41, 0.66) ***	0.56 (0.43, 0.74) ***	1.59 (1.13, 2.22) **	1.44 (1.00, 2.07) *
**Wealth index**				
Poorest	1	1	1	1
Poorer	0.98 (0.72, 1.32)	0.97 (0.72, 1.32)	1.57 (0.90, 2.73)	1.56 (0.89, 2.73)
Middle	1.11 (0.82, 1.49)	1.12 (0.82, 1.52)	1.46 (0.83, 2.56)	1.46 (0.81, 2.60)
Richer	0.92 (0.68, 1.24)	0.92 (0.66, 1.27)	2.01 (1.19, 3.39) **	2.01 (1.14, 3.55) *
Richest	0.92 (0.68, 1.24)	0.97 (0.67, 1.40)	2.17 (1.29, 3.64) **	2.12 (1.14, 3.94) *
**Area of residence**				
Semi-urban	1	1	1	1
Rural	1.09 (0.92, 1.29)	1.07 (0.84, 1.36)	0.77 (0.58, 1.01)	1.04 (0.71, 1.53)
**Health insurance**				
No	1	1	1	1
Yes	1.13 (0.79, 1.62)	0.96 (0.60, 1.52)	1.47 (0.86, 2.52)	1.63 (0.92, 2.87)

¥Adjusted for age categories, wealth index, area of residence and health insurance status; **p<0.05; **p<0.01; ***p<0.001*

Figure S1: Percent distribution of type of facility for outpatient (top panel) and inpatient (bottom panel) service utilization, by household wealth quintile, place of residence and health insurance status

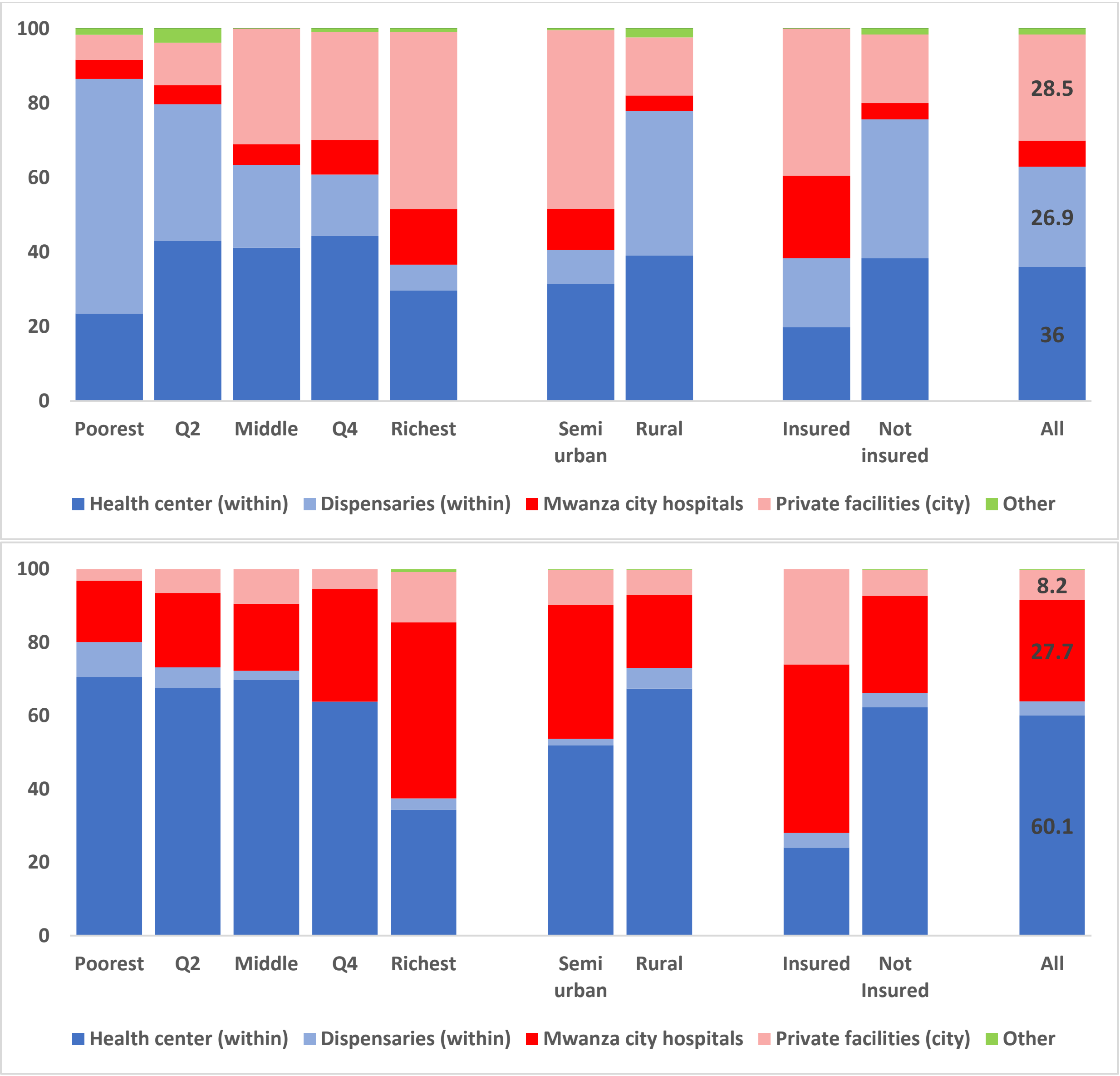

Table S7: Factors associated with outpatient and inpatient utilization in Mwanza city and private health facilities (Crude & Adjusted analysis) among women of reproductive age in Magu Health and Demographic Surveillance site, 2020-2021 (N=8,665)

Characteristic	Outpatient attendance in city hospitals		Inpatient attendance in city hospitals	
	Crude analysis COR (95% CI)	Adjusted analysis¥ AOR (95% CI)	Crude analysis COR (95% CI)	Adjusted analysis¥ AOR (95% CI)
**Age categories**				
15-24	1	1	1	1
25-34	1.46 (1.06, 2.01) *	1.41 (0.95, 2.10)	2.13 (1.62, 2.80) ***	1.97 (1.43, 2.73) ***
35+	1.32 (0.95, 1.83)	1.32 (0.91, 1.93)	1.30 (0.96, 1.76)	1.25 (0.89, 1.76)
**Wealth index**				
Poorest	1	1	1	1
Poorer	1.06 (0.50, 2.26)	0.97 (0.45, 2.08)	1.52 (0.90, 2.57)	1.46 (0.86, 2.48)
Middle	2.72 (1.45, 5.11) **	2.20 (1.15, 4.24) *	1.61 (0.96, 2.70)	1.50 (0.87, 2.56)
Richer	2.86 (1.54, 5.31) **	2.02 (1.03, 3.94) *	2.03 (1.24, 3.32) **	1.79 (1.06, 3.05) *
Richest	5.10 (2.84, 9.14) ***	2.98 (1.51, 5.91) **	3.69 (2.34, 5.82) ***	3.09 (1.79, 5.33) ***
**Area of residence**				
Semi-urban	1	1	1	1
Rural	0.44 (0.33, 0.58) ***	0.66 (0.43, 0.99) *	0.58 (0.46, 0.73) ***	0.89 (0.63, 1.26)
**Health insurance**				
No	1	1	1	1
Yes	5.43 (3.88, 7.61) ***	4.26 (2.81, 6.45) ***	2.65 (1.85, 3.81) ***	2.04 (1.30, 3.20) **

¥Adjusted for age categories, wealth index, area of residence and health insurance status; *$p<0.05$; **$p<0.01$; ***$p<0.001$*

Table S8: Total annual expenditure on health service utilization and self-treatment by household wealth quintile, place of residence and health insurance status among women 15-49 years in Magu Health and Demographic System, 2020-2021

	Service use (TZS)	*Self-treatment (TZS)	Percent on self treatment
**Total**	16,860	23,172	57.9
Age			
15-24	11,859	13,974	54.1
25-34	19,255	27,103	58.5
35+	21,677	32,206	59.8
**Area of residence**			
Semi-urban	19,147	24,239	55.9
Rural	14,702	22,203	60.2
**Wealth status**			
Poorest	11,823	16,504	58.3
Richest	24,510	26,967	52.4
**Health insurance**			
No	16,548	22,931	58.1
Yes	22,490	28,150	55.6
