# Supplementary material for: Universal health coverage for women of reproductive ages: a survey-based comprehensive assessment of service utilisation and health expenditure in Tanzania
Source: BMJ Public Health. 2025 Jan 16;3(1):e000672. doi: 10.1136/bmjph-2023-000672 (PMC11812853; doi:10.1136/bmjph-2023-000672)
Supplement: online supplemental file 4 [file bmjph-3-1-s002.pdf]

NIMR Mwanza
TAZAMA Project
Women Survey Questionnaire

S/N	Question	Response
	Participant's ID<br>UTAMBULISHO WA MSHIRIKI	
**SECTION 1: Background household and individual / MASUALA YA JINSIA, ELIMU, NA SHUGHULI ZA KIUCHUMI**		
101	In what month and year were you born?<br>TAREHE YA KUZALIWA	Month / MWEZI<br><br>Year / MWAKA
**102**	Age: what is your age?<br>UNA UMRI GANI?	Years / MIAKA
**103**	What is your marital status?<br><br>HALI YAKO YA NDOA NI IPI?	1=Single – never married / SIJAWAHI KUOLEWA<br>2=Married / NIMEOLEWA<br>3=Separated /TUMETENGANA<br>4=Divorced / NIMEACHIKA<br>5 = Widow /NI MJANE
104	Have you ever attended school?<br>JE, UMEWAHI KWENDA SHULE?	1=Yes / NDIYO<br>2=No / HAPANA ----------------→ 107
105	If YES: What was the highest level, you attended?<br>KAMA NDIYO: NI KIWANGO GANI CHA JUU CHA ELIMU ULICHOFIKIA?	1=Primary / ELIMU YA MSINGI<br>2=Secondary / ELIMU YA SEKONDARI<br>3=Higher (College/University) / ELIMU YA CHUO<br>4=Others /NYINGINE
106	How many years did you complete at that highest level you achieved?<br>NI MIAKA MINGAPI ULIKAMILISHA KATIKA HIYO NGAZI YA JUU YA ELIMU ULIYOFIKIA?	Number of years ___________<br><br>IDADI YA MIAKA __________
**Residence / MAKAZI**		
107	How long have you been living continuously in this village (NAME OF VILLAGE)? NI KWA MUDA GANI UMEISHI MFULILIZO KATIKA KIJIJI HIKI?	1= Years / MIAKA<br><br>2= Always/born here WAKATI WOTE/NI MZALIWA ----------------→ 109<br><br>3= Visitor / NI MGENI ----------------→ 109
108	Just before you moved to this village, where did you live?<br>KABLA HUJAHAMIA KATIKA KIJIJI HIKI ULIKUWA UNAISHI WAPI?	1 = In this village / KATIKA KIJIJI<br>2 = Other village in Kisesa / VIJIJI VINGINE KISESA<br>3 = Elsewhere in Magu district / MAHALI PENGINE WILAYA YA MAGU<br>4 = Mwanza city / JIJI LA MWANZA<br>5 = Elsewhere in Mwanza region / PENGINE MKOANI MWANZA<br>6 = Elsewhere in Tanzania / PENGINE TANZANIA<br>7 = Other / PENGINE___________
**WATER & SANITATION / MAJI NA CHOO**		
109	What is the main source of drinking water for you and the other members of your household?<br>NI KIPI CHANZO KIKUU CHA MAJI YA KUNYWA KWA AJILI YAKO NA WANAKAYA WENGINE?	**PIPED WATER / MAJI YA BOMBA**<br>1= Piped into dwelling / MAJI YA BOMBA KWENYE KAYA<br>2= Piped to yard or plot / MAJI YA BOMBA UWANJA<br>3=Piped to neighbor / MAJI YA BOMBA KWA JIRANI<br>4=Public tap/standpipe -BOMBA LA JAMII<br><br>5 = Tube well or borehole / KISIMA

		**DUG WELL / KISIMA KILICHOCHIMBWA**<br>6 = Protected well / KISIMA KILICHOFUNIKWA<br>7 = Unprotected well/ KISIMA KISICHOFUNIKWA<br><br>**WATER FROM SPRING / MAJI KUTOKA KWENYE CHEMU CHEMU**<br>8= Protected spring / KISIMA KILICHOFUNIKWA<br>9 = Unprotected spring / KISIMA KISICHOFUNIKWA<br><br>10 = Rain water / MAJI YA MVUA<br>11 = Tanker truck / MAJI YA TANKI LA GARI<br>12 = Cart with small tank<br><br>13 = Surface water (river/dam/ lake/pond/stream) / MAJI YA ZIWA, MTO, YA UMWGILIAJI<br>14 = Bottled water / MAJI YA CHUPA<br>15= Other
110	What kind of toilet facility does your household use? MNATUMIA AINA GANI YA CHOO KATIKA KAYA HII?	**FLUSH OR POUR FLUSH TOILET / CHOO CHA KUFALSHI**<br>1 = Flush to piped sewer system /CHOO CHA KUFLASHI KWENDA KWENYE MFUMO<br>2 = Flush to septic tank / CHOO CHA KUFLASHI KWENDA KWENYE SEPTIKI TANKI<br>3 = Flush to pit latrine / KUFLASHI KWENDA KWENYE CHOO CHA SHIMO<br>4 = Flush to somewhere else / KUFLASH KWENDA MAHALA PENGINE<br>5 = flush, don't know where / KUFLASHI BILA KUJUA VINAKWENDA<br><br>**PIT LATRINE / CHOO CHA SHIMO**<br>6 = Ventilated improved pit latrine / CHOO CHA SHIMO CHENYE BOMBA LA HEWA<br>7 = Pit latrine with slab / CHOO CHA SHIMO CHENYESILABU<br>8= Pit latrine without slab/open pit / CHOO CHA SHIMO BILA SILABU<br><br>9 = Bucket toilet / CHOO CHA NDOO<br><br>10 = No facility/bush/field / HAKUNA CHOO<br><br>11= Other / NYINGINE
111	Do you share this toilet facility with other households?<br>JE UNASHIRIKIANA CHOO HIKI NA KAYA ZINGINE?	1 = Yes / NDIYO<br>2 = No / HAPANA
112	Do you own a mobile phone? If yes is it a smart phone?<br>JE UNAMILIKI SIMU YA MKONONI? JE NI SIMU JANJA?	1 = Yes, I own a smart phone/NDIYO, NINA MILIKI SIMU JANJA<br>2 = Yes, I own a phone but not a smart phone/NDIYO NINA MILIKI SIMU LAKINI SIYO SIMU JANJA<br>3= I don’t own a phone, but share one in the household/SIMILIKI SIMU LAKINI NINATUMIA NA WANAFAMILIA KWENYE KAYA<br>4 = No/HAPANA -→ Go to the next section
113	Have you used your phone in the last week?<br>JE ULITUMIA SIMU YAKO WIKI ILIOPITA?	1 = Yes/NDIYO<br>2 = No/HAPANA
114	In the last 12 months, have you used a mobile phone to make financial transactions such as sending or receiving money, paying bills, purchasing goods or services, or receiving wages? JE KATIKA KIPINDI CHA MIEZI 12 ILIYOPITA UMESHAITUMIA SIMU	1 = Yes / NDIYO<br>2 = No / HAPANA

	YAKO KUFANYA MIAMALA KAMA KUTUMA NA KUPOKEA PESA, KULIPIA BILI, KUNUNUA BIDHAA AU HUDUMA?	

Section 200
Child survival and pregnancy history / WATOTO NA UHAI WAO
Now I would like to ask about all the births you have had during your life.
SASA NINGEPENDA KUKUULIZA MASWALI KUHUSU WATOTO WAKO ULIOWAZAA MWENYEWE HADI SASA

201	Have you ever given birth?<br>JE UMESHAWAHI KUJIFUNGUA MTOTO?<br><br>CHECK: ASK THE WOMAN WHO HAS NEVER GIVEN BIRTH<br>MUULIZE MWANAMKE AMBAYE HAJAWAHI KUJIFUNGUA?<br><br>Have you had a baby who has died shortly after being born?<br>JE UMESHAWAHI JIFUNGUA MTOTO AMBAYE ALIFARIKI MDA MCHACHE TUU BAADA YA KUJIFUNGUA?	1 = Yes /NDIYO<br>2 = No /HAPANA-----→ Ask check question:<br><br>If check question is:<br>Yes ---→ Skip to Q 204<br>No --→ Skip to Q 301
202	Do you have any sons or daughters to whom you have given birth who are now living with you? How many?<br>JE UNA MTOTO WA KIUME AU WA KIKE AMBAO UMEWAZAA NA WANAISHI NA WEWE? NI WANGAPI?<br><br>WHAT ARE THEIR NAMES_______________<br>TAJA MAJINA YAO______________<br><br>IF NONE RECORD “00”<br>KAMA HANA MTOTO ANDIKA “00”	Number /IDADI__________<br>00= None/HAKUNA<br><br>For each son & daughter<br>KWA KILA MTOTO WA KIUME NA WA KIKE<br><br>First name________Second name________<br>JINA LA KWANZA_____ JINA LA PILI_______<br><br>First name________Second name________<br>JINA LA KWANZA______ JINA LA PILI________
203	Do you have any sons or daughters to whom you have given birth who are now living elsewhere? How many?<br>JE UNA MTOTO WA KIUME AU WA KIKE AMBAO UMEWAZAA NA WANAISHI SEHEMU NYINGINE MBALI NA WEWE? NI WANGAPI?<br><br>WHAT ARE THEIR NAMES _____________<br>TAJA MAJINA YAO________________<br>IF NONE RECORD “00”<br>KAMA HANA ANDIKA “00”	Number/IDADI __________<br>00= None /HAKUNA<br><br>For each son & daughter<br><br>First name________Second name________<br>JINA LA KWANZA_____ JINA LA PILI_______<br><br>First name________Second name________<br>JINA LA KWANZA______ JINA LA PILI________
204	Have you ever given birth to a boy or girl who was born alive but later died (at any age)?<br>JE UMESHAWAHI KUJIFUNGUA MTOTO (MVULANA/MSICHANA) AMBAE ALIKUWA HAI NA BAADAE AKAFARIKI?	Number/IDADI _______<br>00= None/HAKUNA<br><br>For each child who died<br>KWA KILA MTOTO ALIEFARIKI

<table>
<tr><td></td><td>WHAT ARE THEIR NAMES________________
TAJA MAJINA YAO___________________

For all women PROBE: Have you given birth to any baby who cried, who made any movement, sound, or effort to breathe, or who showed any other signs of life even if for a very short time?
KAMA HAKUNA, JARIBU KUDADISI KWA KUMUULIZA KAMA AMESHAWAHI KUJIFUNGUA MTOTO ALIELIA, AU KUONESHA ISHARA ZA UHAI KWA KUHEMA, KUTOA MLIO/SAUTI KWA MDA MFUPI</td><td>First name________Second name________
JINA LA KWANZA_____ JINA LA PILI_______

First name________Second name________
JINA LA KWANZA______ JINA LA PILI________
….
If child did not have a name, then write BABY1 and the mothers name as the Second name,
KAMA MTOTO HANA JINA, ANDIKA BABY1KAMA JINA LA KWANZA NA JINA LA MAMA KAMA JINA LA PILI
And then BABY2 if a second baby did not have a name, etc.
NA PIA BABY2 KAMA MTOTO WA PILI HAKUWA NA JINA NA N. K</td></tr>
<tr><td>205</td><td>CHECK The total number of births which is Q 202 + Q203 + Q204
ANGALIA IDADI KAMILI YA UZAO WA MAMA KWA KUJUMLISHA Q202+Q203+Q204
Just to make sure I have this right, you have had in total __ births during your life time. Is that correct? IF NOT CORRECT
HOW MANY CHILDREN HAVE WE MISSED? _____ (IF NO MISSED CHILDREN WRITE 00)

KWA KUHAKIKI TAARIFA YAKO, UMEZAA JUMLA YA WATOTO __________ HADI SASA. KAMA SIYO KWELI
JE, KUNA WATOTO WANGAPI TUMEKOSA KUANDIKA?
(KAMA HAKUNA ANDIKA “00”)</td><td>
How many missed children: ______
JE, KUNA WATOTO WANGAPI TUMEKOSA KUANDIKA?

For each missed child

First name________Second name________
JINA LA KWANZA_____ JINA LA PILI_______

First name________Second name________
JINA LA KWANZA______ JINA LA PILI________</td></tr>
<tr><td>206</td><td>
Now add all the children again
SASA JUMLISHA IDADI YA WATOTO WOTE TENA
Q202 + Q203 + Q204 + Q206 = _____
Is this right?
IDADI YA WATOTO IPO SAHIHI?

If no births go to section 300
KAMA HANA UZAO NENDA SECTION 300</td><td>

Total number of births _______________

JUMLA YA KIZAZI _________________________

If no births-> skip to section 300
KAMA HANA UZAO NENDA SECTION 300</td></tr>
<tr><td></td><td colspan="2">For each child (name) complete questions 213-219
note: all children are named in q202, q203, q204 and q206 in the order that was listed by the mother. those who died will come last.
JIBU MASWALI 213 -219 KWA KILA JINA LA MTOTO.
NOTE: LISTI YA MAJINA YA WATOTO INAPATIKANA KWENYE MASWALI 202,203,204 NA 206 KAMA ILIVYOTOLEWA NA MAMA MZAZI WA WATOTO HAO.KWA WATOTO WALIOFARIKI WATAKUWA WAMWISHO KWENYE LISTI</td></tr>
</table>

207-01	Is NAME a boy or girl?<br>JE NI MVULANA AU MSICHA?	1= Boy / MVULANA<br>2=Girl / MSICHANA
208-01	Was it a single or multiple birth?<br>JE ULIKUWA UZAZI WA MTOTO MMOJA AU MAPACHA?	1=Single CHILD / MTOTO MMOJA<br>2=Multiple BIRTH / MAPACHA
209-01	On what month and year was (NAME) born?<br>JE MTOTO HUYU ALIZALIWA MWEZI NA MWAKA GANI?	Month / MWEZI ________<br>Year / MWAKA _________
210-01	Is (NAME) still alive?<br><br>JE JINA BADO YUPO HAI?	1=Yes / NDIYO<br>2=No / HAPANA -----→ Q218
211-01	How old was (NAME) at his last birthday?<br>JE JINA ALIKUWA NA UMRI WA MIAKA MINGAPI KATIKA SIKU YAKE YA KUZALIWA YA MARA YA MWISHO?	Age (in completed years)<br><br>UMRI KATIKA MIAKA ILIYOKAMILIKA _______<br>RECORD 00 IF LESS THAN 1 YEAR<br>ANDIKA 00 KAMA NI CHINI YA MWAKA MMOJA
212-01	Is NAME living with you?<br><br>JE JINA ANAISHI NA WEWE?	1=Yes / NDIYO<br><br>2= No elsewhere in Kisesa DSS<br>KWINGINE NDANI YA ENEO LA SENSA (DSS)→ Go TO THE NEXT BIRTH<br><br>3= No outside DSS area<br>HAPANA NJE YA ENEO LA SENSA (DSS) → Go TO THE NEXT BIRTH
213-01	Has (NAME) been sick in the last 4 weeks?<br>JE JINA AMEUGUA KATIKA KIPINDI CHA WIKI NNE ZILIZOPITA?	1= Yes / NDIYO<br>2=No / HAPANA---→ Go to the next birth
214-01	What was the problem?<br>SHIDA ILIKUWA NI NINI?<br><br>Any other issues?<br>KUNA SHIDA NYINGINE?<br><br>CIRCLE ALL THAT WERE MENTIONED<br>ZUNGUSHIA SHIDA ZOTE ZILIZOTAJWA	A = Fever / MALARIA<br>B = Diarrhoea/KUAHARISHA<br>C = Cough/KUKOHOA<br>D = Difficult breathing/KUPUMUA KWA SHIDA<br>E = Injury/ MAJERAHA<br>F = Skin disease/UGONJWA WA NGOZI<br>G = Other/NYINGINE ____________
215-01	Did you give (NAME) any treatment at home?<br>JE (JINA) AMEPATIWA MATIBABU YOYOTE NYUMBANI?<br>Which kind of treatment?<br>NI MATIBABU YA AINA GANI?	1 = Medicines /MADAWA<br>2 = Other treatment/MATIBABU MENGINE<br>3 = No /HAPANA
216-01	Did you take him/her to a health facility?<br>JE ULIMPELEKA KWENYE KITUO CHA AFYA/ZAHANATI?	1= Yes / NDIYO<br>2=No / HAPANA --→ Go to the next birth
217-01	IF YES: which health facility?<br>KAMA NI NDIYO, NI HOSPITALI GANI (YAANI ZAHANATI/KITUO CHA AFYA/HOSPITALI)?	1= Kisesa health centre<br>2= Igekemaja disp<br>3= Welamasonga disp

		4= Isangijo disp.<br>5= Kanyama disp<br>6=Ihayabuya disp<br>10= Sekou toure<br>11= Bugando medical centre<br>12=Sumve hospital<br>13=Magu hospital<br>14= Private health facility<br>15= Other hospital or health facility<br>------------------------→ Go to the next birth
218-01	How old was NAME when he/she died? (RECORD DAYS if LESS THAN 1 MONTH; MONTHS IF LESS THAN 2 Years; or Years)<br><br>JE JINA ALIKUWA NA UMRI WA GANI ALIPOFARIKI?	1= Days / SIKU<br>2 = Months / MWEZI<br>3 = Years /MWAKA<br><br>NUMBER / IDADI YA SIKU, MIEZI AU MIAKA __ _<br><br>----→ Go to the next birth
219	Have you ever had a pregnancy that ended in a stillbirth where did the baby not cry, did not move or breathe at all?<br>JE, UMESHAWAHI KUWA NA MIMBA AMBAYO ULIJIFUNGUA MTOTO MFU?	1= Yes / NDIYO<br>2 = No / HAPANA ----→ Go to section 300
220	How long ago did this last such pregnancy end?<br><br>NI MUDA GANI UMEPTITA TANGU ULIPOPATA TUKIO LA KUJIFUNGUA MTOTO MFU?	1=Years ago<br>Record 00 if less than 1 year ago<br>RECORD 96 IF Do not remember/don't know<br>96= SIKUMBUKI
221	IF LESS THAN 6 YEARS AGO: How many months did this pregnancy last?<br>KAMA NI CHINI YA MIAKA 6 ILIYOPITA, JE MIMBO HIYO ILIKUWA NA MIEZI MINGAPI?	1= Months of pregnancy / MIEZI YA UJAUZITO________<br>96 = Don't remember / SIKUMBUKI

SECTION 300: Service utilization: general<br>MATUMIZI YA HUDUMA ZA AFYA: MAMA<br>Now I would like to ask about your health and your use of health services.<br>Please tell us about your own experience with health services and not about the health services you used for your children (that will come later)<br>SASA NINGEPENDA KUKUULIZA MASWALI KUHUSU AFYA YAKO BINAFSI NA MATUMIZI BINAFSI YA HUDUMA ZA AFYA.		
301	How is your health today?<br><br>AFYA YAKO LEO IKO JE?	1= Very good / NZURI SANA<br>2= Good / NZURI<br>3= Average / WASTANI<br>4= Poor/ MBAYA<br>5= Very poor/ MBAYA SANA
302	Do you have any limitations in normal daily activities because of your health?<br><br>JE, UNA KIZUIZI CHOCHOTE KATIKA SHUGHULI ZAKO ZA KILA SIKU KUTOKANA NA HALI YA AFYA YAKO?	1= Yes / NDIYO<br>2= No / HAPANA<br>98= Don`t know/SIJUI
303	Do you have health insurance?<br><br>JE, UNA BIMA YA AFYA?	1= Yes / NDIYO<br>2= No / HAPANA -------------------------→ 307<br>98= Don`t know / 8=SIJUI ---→ 307
304	What type of health insurance?<br><br>NI AINA GANI YA BIMA ULIYONAYO?	1 = Community health insurance / BIMA YA JAMII<br>2=National health insurance / BIMA YA AFAYA YA TAIFA<br>2= Employer-based insurance / BIMA YA MWAJIRI<br>3= Social security / BIMA YA ULINZI WA JAMII<br>4= Other private insurances / AINA NYINGINE YA BIMA BINAFSI
305	Is it a family or household insurance or your individual insurance?<br>JE NI BIMA YA KAYA AU NI YA BINAFSI?	1 = Family or household/FAMILIA AU KAYA<br>2 = Individual/MTU BINAFSI
306	How much does your health insurance cost per year?<br>JE, BIMA YA AFYA INA GHARAMA GANI KWA MWAKA?	Cost of health insurance per year in TSH __________________<br>GHARAMA YA BIMA YA AFAYA KWA MWAKA _______
307	During the last 12 months, were you admitted to a hospital?<br><br>KATIKA KIPINDI CHA MIEZI 12 ILIYOPITA UMELAZWA HOSPITALI?	1= Yes / NDIYO<br>2= No / HAPANA ------------------------→ 315<br>98= Don`t know / SIJUI ---→ 315
308	How many times were you admitted (stayed overnight) to a hospital during the last 12 months?<br>JE KATIKA MIEZI 12 ILIYOPITA NI MARA NGAPI ULILAZWA HOSPITALINI?	Number of times ________<br><br>MARA NGAPI ___________
309	For the last admission: how many months ago were you admitted?	1=Months ago ______<br>1= IDADI YA MIEZI ILIYOPITA ____

	KWA ULIPOLAZWA HOSPITALI MARA YA MWISHO: NI MIEZI MINGAPI IMEPITA?	2= If less than one month ago put 00<br>KAMA CHINI YA MWEZI MMOJA WEKA 00
310	Where were you admitted (the last admission)?<br><br>ULILAZWA KATIKA HOSPITALI GANI?	1= Kisesa health centre<br>2= Igekemaja disp<br>3= Welamasonga disp<br>4= Isangijo disp.<br>5= Kanyama disp<br>6=Ihayabuya disp<br>10= Sekou toure<br>11= Bugando medical centre<br>12=Sumve hospital<br>13=Magu hospital<br>14= Private health facility<br>15= Other hospital or health facility
311	How many nights did you spend in the hospital?<br><br>ULILALA HOSPITALI KWA SIKU NGAPI?	Number of nights / ULILALA SIKU NGAPI? ___________
312	What was the main reason for seeking care this most recent hospital admission?<br><br>NINI ILIKUWA SABABU KUU YA WEWE KUHITAJI MATIBABU KWA HII MARA YA MWISHO ULIPOLAZWA HOSPITALI?	1= Pregnancy / UJAUZITO<br>2= Illness / NILIKUWA MGONJWA<br>3= Injury/Accident / NILIPATA AJALI<br>4= Other / NYINGINE<br>5 = Her child was admitted
313	How much money did you or the family spend on treatment and services for your admission (stay) in hospital?<br>NI KIASI GANI CHA FEDHA (KWA UJUMLA) WEWE AU FAMILIA ILITUMIA KATIKA MATIBABU YAKO ulipolazwa hospitalini?	Total cost of hospital admission TSH _________<br><br>JUMLA YA GHARAMA YA MATIBABU TZS _________
314	How satisfied with the health services?<br><br>ULIRIDHIKA KIASI GANI NA HUDUMA ZA AFYA ULIZOPATIWA?	1=Very satisfied/NILIRIDHIKA SANA<br>2= Satisfied/NILIRIDHIKA<br>3 = Not satisfied / SIKURIDHIKA<br>4= Not satisfied at all/SIKURIDHIKA HATA KIDOGO
315	In the last 4 weeks, did you use any other health facility for treatment/services for your own health (not staying overnight)?<br><br>KATIKA KIPINDI CHA WIKI NNE ZILIZOPITA, UMEKWENDA KATIKA HOSPITALI (ZAHANATI/KITUO CHA AFYA/HOSPITALI) KWA AJILI YA MATIBABU AU HUDUMA BILA KULAZWA?	1= Yes / NDIYO<br>2= No / HAPANA ----------------------→322
316	How many times did you visit a health facility for services in the last 4 weeks?<br><br>KATIKA WIKI NNE ZILIZOPITA, NI MARA NGAPI ULIKWENDA HOSPTALI KWA AJILI YA MATIBABU AU HUDUMA?	Number of times ______<br>MARA NGAPI_________
317	How many days ago did you make this last visit?	Days ago __________

	NI SIKU NGAPI ZIMEPITA TANGU UENDE HOSPITALI KWA MARA YA MWISHO?	SIKU NGAPI ZILIZOPITA ____
318	Where did you go for services?<br><br>ULIKWENDA KWENYE KITUO KIPI CHA AFYA KUPATA HUDUMA?	1= Kisesa health centre<br>2= Igekemaja disp<br>3= Welamasonga disp<br>4= Isangijo disp.<br>5= Kanyama disp<br>6=Ihayabuya disp<br>10= Sekou toure<br>11= Bugando medical centre<br>12=Sumve hospital<br>13=Magu hospital<br>14= Private health facility<br>15= Other hospital or health facility
319	What was the main reason for the visit?<br><br>NINI ILIKUWA SABABU KUU YA WEWE KWENDA HOSPITALI KWA MATIBABABU/HUDUMA	1= Pregnancy/ UJAUZITO<br>2= Delivery care/ KUJIFUNGUA<br>3= Immunization (Preventive child care)/ CHONJO KWA MTOTO<br>4= Family planning/ UZAZI WA MPANGO<br>5= Illness/ KUUMWA<br>6= Injury/ MAJEREHA<br>7= Accident/AJALI<br>8= Other/ NYINGINE (TAJA)
320	How much money did you (or your family) spend on treatment and services for your last visit to the health facility, including medicines?<br><br>NI KIASI GANI CHA FEDHA ULICHOTUMIA AU FAMILIA WAKATI ULIPOENDA HOSPITALI MARA YA MWISHO KWA MATIBABU AU HUDUMA NA DAWA?	Total amount spent TSH _______<br><br>JUMLA YA FEDHA ZA MATIBABU _______
321	How satisfied with the health services?<br><br>ULIRIDHIKA KWA KIWANGO GANI NA HUDUMA ULIYOPATIWA?	1=Very satisfied /NILIRIDHIKA SANA<br>2= Satisfied / NILIRIDHIKA<br>3 = Not satisfied / SIKURIDHIKA<br>4= Not satisfied at all/<br>SIKURIDHIKA HATA KIDOGO
322	In the last 4 weeks, did you spend any money on medicines or other kinds of treatment that you bought yourself?<br>KATIKA KIPINDI CHA WEEK 4 ZILIZOPITA UMESHAWAHI KUTUMIA FEDHA YAKO BINAFSI KWENYE MADAWA AU MATIBABU YA AFYA?	Total amount spent TSH _______<br><br>JUMLA YA FEDHA ZA MATIBABU _______
323	Has a doctor or nurse ever examined you for cervical cancer?<br><br>JE, MGANGA AU MUUGUZI AMEWAHI KUKUFANYIA UCHUNGUZI WA	1=Yes/NDIYO<br>2=No/HAPANA ------------------------------→325

	MAAMBUKIZI YA KANSA YA SHINGO YA UZAZI?	
324	When was the last time?<br>LINI ILIKUWA MARA YA MWISHO?<br>Last time checked (months ago) _____<br><br>LINI ULICHUNGUZWA KWA MARA YA MWISHO __________	Months ago/MIEZI MINGAPI ILIYOPITA__________
325	Have you ever received a vaccine for prevention of cervical cancer (HPV vaccination)?<br><br>JE UMEWAHI KUPATA CHANJO KWA AJILI YA KUZUIA KANSA YA SHINGO YA KIZAZI?	1= Yes / NDIYO<br>2=No / HAPANA<br>98=Don’t know / SIJUI

Section 400 Family planning<br>UZAZI WA MPANGO<br>Now I would like to ask you about family planning methods<br>SASA NINGEPENDA KUKUULIZA MASWALI MACHACHE YANAYOHUSIANA NA MATUMIZI YA NJIA ZA UZAZI WA MPANGO			
400	Are you currently pregnant?<br>JE KWA SASA WWEWE NI MJAMZITO?	1=Yes / NDIYO----------→ 500<br>2=No / HAPANA	
401	Are you currently using any method to delay or avoid getting pregnant?<br>JE WEWE UNATUMIA NJIA ZA UZAZI WA MPANGO KWA SASA?	1=Yes / NDIYO<br>2=No / HAPANA ----------→ 403	
402	Which method are you using?<br><br>SELECT ALL THE MENTION METHODS<br><br>NI NJIA GANI YA UZAZI WA MPANGO UNAYOTUMIA?<br><br>CHAGUA NJIA ZOTE ATAKAZOKUTAJIA	A=Female sterilization / KUFUNGA KIZAZI MWANAMKE<br>B=Male sterilization / KUFUNGA KIZAZI MWANAME<br>C=IUD<br>D=Injectable / SINDANO<br>E=Implants / NJITI<br>F=Pills / VIDONGE<br>G=Male condom / KONDOMU YA KIUME<br>H=Female condom / KONDOMU YA KIKE<br>I= Rhythm<br>J = Lactational Amerrhoea Method<br>K = Other Traditional methods / NJIA NYINGINE ZA KIENYEJI<br>L= Other / NYINGINE___________	
403	Is your partner currently using any method to delay or avoid getting pregnant?<br><br>JE MWENZI WAKO KWA SASA ANATUMIA NJIA YOYOTE YA UZAZI WA MPANGO KUEPUKA AU KUZUIA KUPATA UJAUZITO?	1=Yes / NDIYO<br>2=No / HAPANA<br><br>IF 401 = NO AND 403 = NO<br>SKIP ----------→ 500	
404	Which method is he using?<br>ANATUMIA NJIA GANI?	B. Male sterilization/KUFUNGA KIZAZI MWANAUME<br>G. Male condoms/KONDOMU ZA KIUME<br>K. Other traditional methods/NJIA NYINGINE ZA KIENYEJI<br>L. Other/NYINGINE TAJA	
405	Where did you obtain the method you OR YOUR PARTNER are using?<br>JE NJIA YA UZAZI WA MPANGO UNAYOTUMIA ULIPATA WAPI?	1= Kisesa health centre<br>2= Igekemaja disp<br>3= Welamasonga disp<br>4= Isangijo disp.<br>5= Kanyama disp<br>6=Ihayabuya disp<br>10= Sekou toure	

		11= Bugando medical centre<br>12=Sumve hospital<br>13=Magu hospital<br>14= Private health facility<br>15= Other hospital or health facility<br>20 Pharmacy/DUKA LA DAWA<br>21 Kiosk/KIOSKI<br>22= Other shop/DUKA	
406	How easy is it for you to get the modern contraceptives you want?<br>KUNA URAHISI KIASI GANI KATIKA KUPATA NJIA ZA KISASA ZA UZAZI WA MPANGO?	1 = Easy/NI RAHISI<br>2 =Somewhat difficult/ NI VIGUMU KIDOGO<br>3 = Very difficult/NI VIGUMU SANA<br>4 = Don`t want any/SIHITAJI NJIA YOYOTE<br>98 = Don't know/SIJUI	
407	Do you pay for your contraception? If so how much?<br>JE UNALIPIA HUDUMA YA UZAZI WA MPANGO?<br>KAMA NI NDIO, UNALIPIA SHILINGI NGAPI?	Amount/KIASI_____<br>PUT 00 IF NONE	

Section 500
Antenatal and delivery care
HUDUMA ZA UJAUZITO NA KUZAA

Now I would like to ask questions about Antenatal and delivery services you received during pregnancy
SASA NINGEPENDA KUKUULIZA MASWALI MACHACHE KUHUSU HUDUMA ZA AFYA YA MAMA NA MTOTO ULIZOPATA WAKATI WA UJAUZITO

500	FILTER: Respondent has a birth(s) after September 1, 2017<br><br>MCHUJO: MHOJIWA ANA MTOTO AU WATOTO WALIOZALIWA BAADA YA SEPTEMBER 1, 2017<br><br>AUTO FILL the NAME of all births under the age of 3<br>AUTOMATIKALE JAZA MAJINA YA WATOTO WOTE WENYE UMRI WA CHINI YA MIAKA 3<br><br>AUTO FILL the MONTH and YEAR OF the BIRTHS<br><br>AUTOMATIKALE JAZA MWEZI NA MWAKA WA KUZALIWA MTOTO	First name________Second name________<br>JINA LA KWANZA_____ JINA LA PILI_______<br><br>First name________Second name________<br>JINA LA KWANZA______ JINA LA PILI________<br><br>(NAME)Birth month___________<br>MWEZI AMBAO(JINA) ALIZALIWA_________<br>(NAME)Birth year______________<br>MWAKA AMBAO (JINA) AMEZALIWA_______<br><br>If no child/children born after September 1 2017 -→ Skip to 600<br>KAMA HAKUNA MTOTO/WATOTO WALIOZALIWA BAADA YA TAREHE 1 MWEZI WA TISA 2017→RUKA KWENDA SECTION 600
	ASK all questions in both sections 500 and 600 for each completed pregnancy<br>Note: Start with the former completed pregnancy and end up with the most current completed pregnancy<br>ULIZA MASWALI YOTE YA 500 NA 600 KWA KILA UJAUZITO ALIOBEBA MAMA KWA KIPINDI CHA BAADA YA TAREHE 1 MWEZI WA TISA 2017<br>ANZA NA UJAUZITO WA KWANZA NA UMALIZIE NA UJAUZITO WAKE WA MWISHO	
501	When you were carrying(Name), did you receive antenatal care from a health care provider?<br>WAKATI UMEBEBA MIMBA YA (JINA), ULIPATA HUDUMA YA KLINIKI YA WAJAWAZITO KUTOKA KWA MTUMISHI WA AFYA?	1= Yes / NDIYO<br>2= No / HAPANA ---------------------→510
502	Where did you go for antenatal care?<br><br>ULIKWENDA WAPI KWA AJILI YA HUDUMA KLINIKI YA WAJAWAZITO?<br><br>List all the places that she went to	1= Kisesa health centre<br>2= Igekemaja disp<br>3= Welamasonga disp<br>4= Isangijo disp.<br>5= Kanyama disp<br>6=Ihayabuya disp<br>10= Sekou toure<br>11= Bugando medical centre<br>12=Sumve hospital<br>13=Magu hospital

		14= Private health facility<br>15= Other hospital or health facility
503	How many months were you pregnant when you went for the first antenatal visit?<br><br>JE UJAUZITO WAKO ULIKUWA WA MIEZI MINGAPI ULIPOKWENDA KWA MARA YA KWANZA KLINKI YA WAJAWAZITO?	Pregnancy months ____________<br>UMRI WA UJAUZITO KATIKA MIEZI________
504	How many visits did you make for antenatal care?<br><br>ULIKWENDA MARA NGAPI KLINIKI YA WAJAWAZITO KWA UJAUZITO HUO WA (JINA)?	Number of visits _______________<br>IDADI YA MAHUDHURIO __________
505	Were you offered a test for HIV as part of your antenatal care?<br><br>WAKATI UNAHUDHURIA KLINIKI YA UJAUZITO ULISHAURIWA KUPIMA VVU?	1= Yes / NDIYO<br>2=No / HAPANA ------------------→ 507<br>98=Don't know / SIJUI
506	I don't want to know the results, were you tested for HIV as part of your antenatal care?<br>SITAHITAJI<br><br>KUJUA MAJIBU LAKINI ULIPIMWA VVU ULIPO HUDHURIA KLINIKI?	1= Yes / NDIYO<br>2=No / HAPANA<br>98=Don't know / SIJUI
507	Were you screened for syphilis as part of your antenatal care?<br><br>WAKATI UNAHUDHURIA KLINIKI, ULIPIMA KASWENDE?	1=Yes/NDIO<br>2=No/HAPANA<br>98=Don`t know/SIJUI
508	Were you checked for hemoglobin level during your Antinental visits?<br><br>JE ULIPIMWA KIWANGO CHA DAMU MWILINI WAKATI UNAHUDHURIA KLINIKI?	1=Yes/NDIO<br>2=No/HAPANA<br>98=Don`t know/SIJUI
509	Was your blood pressure checked as part of Antinental care?<br>JE ULIPIMWA SHINIKIZO LA DAMU KAMA HUDUMA YA KLINIKI YA MAMA NA MTOTO?	1=Yes/NDIO<br>2=No/HAPANA<br>98=Don`t know/SIJUI
510	During this pregnancy, did you take any iron tables or iron syrup?<br>KATIKA UJAUZITO HUU WA(JINA), ULITUMIA MADINI JOTO KAMA VIDONGE AU SYRUPU?	1= Yes / NDIYO<br>2=No / HAPANA------------------------→ 512<br>2=HAPANA<br>98=Don't know / SIJUI
511	If yes: for how many days did you take iron?<br><br>KAMA NDIYO: ULITUMIA KWA SIKU NGAPI?	Days taken iron tablets _______<br><br>MUDA WA KUTUMIA MADINI JOTO ____
512	During this pregnancy did you take medicines (SP/Fansidar) to prevent you from getting malaria?	1= Yes / NDIYO<br>2=No / HAPANA ------------------→ 514<br>98=Don't know / SIJUI

	KATIKA KIPINDI CHA MIMBA HIYO, ULITUMIA DAWA YA MALARIA (FANSIDAR)	
513	IF Yes: How many times did you take SP/Fansidar?<br><br>KAMA NDIYO: HIYO FANSIDAR ULITUMIA MARA NGAPI?	Number of times take Fansidar______<br><br>MARA NGAPI KATUMIA FANSIDAR _____
514	Where did you give birth?<br><br>ULIJIFUNGULIA WAPI?	1= Kisesa health centre<br>2= Igekemaja disp<br>3= Welamasonga disp<br>4= Isangijo disp.<br>5= Kanyama disp<br>6=Ihayabuya disp<br>10= Sekou toure<br>11= Bugando medical centre<br>12=Sumve hospital<br>13=Magu hospital<br>14= Private health facility<br>15= Other hospital or health facility<br>31=At home ---------------------→NEXT SECTION<br>32=At Traditional birth attendant/MKUNGA -----------------→NEXT SECTION<br>33=At traditional healer place/MGANGA -→ NEXT SECTION<br>34=Other place (NOT A HOSPITAL)
515	Was the baby delivered by C-section (operation) or the normal way?<br><br>JE (JINA) ALIZALIWA KWA NJIA YA KUPASULIWA AU NJIA YA KAWAIDA?	1= Delivered normally / ALIZALIWA KWA NJIA YA KAWAIDA<br>2=Delivered by C-section / ALIZALIWA KWA KUPASULIWA

SECTION 600 Child Health – only for women with birth/births after Sept 1 2017:

AFYA YA MTOTO: KWA WANAWAKE WENYE WATOTO WALIOZALIWA BAADA YA SEPT 1, 2017 (yaani watoto chini ya miaka 3)		
600	FOR EACH CHILD (NAME) COMPLETE QUESTIONS 600-609<br>NOTE: ALL CHILDREN ARE NAMED IN Q500 IN THE ORDER THAT WAS LISTED BY THE MOTHER. FOR TWINS THE QUESTIONS WILL CONTINUE TO BE ASKED FOR ONLY ONE CHILD.<br><br>FILTER: INTERVIEWER Continue with the child/children in the previous section (born after Sep 1 2017),<br><br>MCHUJO: MHOJAJI ENDELEA NA MTOTO KUTOKA SEHEMU ILIYOTANGULIA (MTOTO AMEZALIWA BAADA YA SEPTEMBA 1, 2017?<br><br>AUTO FILL BY PROGRAM<br><br>AUTOMATIKALE JAZA PROGRAMU	
601	Do you have a child health card or other record where the vaccinations of (NAME) are written down?<br><br>JE, UNA KADI YA AFYA YA MTOTO AU AINA NYINGINNE YA REKODI/KUMBUKUMBU AMBAPO TAARIFA ZA CHANJOYA MTOTO ZIMEANDIKWA?	1 = Yes / NDIYO<br>2 = No / HAPANA --→606
602	IF YES: Can I see it:<br>KAMA NDIYO, NAWEZA KUIONA.	1=Card seen / KADI IMEONEKANA<br>2= Card not seen / KADI HAIJAONEKANA -------→ 606
602A	BCG	Number of doses _______<br>IDADI YA DOZI______<br>00 None/HAKUNA
602B	OPV	Number of doses _______<br>IDADI YA DOZI<br>00 None/HAKUNA
602C	DTP-HepB-Hib	Number of doses ______<br>IDADI YA DOZI<br>00 None/HAKUNA
602D	PCV	Number of doses ______<br>IDADI YA DOZI<br>00 None/HAKUNA
602E	Rota	Number of doses _____<br>IDADI YA DOZI

		00 None/HAKUNA
602F	Surua - rubella	Number of doses_______<br>IDADI YA DOZI<br>00 None/HAKUNA
603	Was birth weight documented in the child health card?<br>JE UZITO WA MTOTO UMEREKODIWA KWENYE KADI YA MAENDELEO YA UKUAJI WA MTOTO?	1 = Yes / NDIYO<br>2 = No / HAPANA --→605
604	If yes, what was the birth weight?<br><br>KAMA NI NDIO, UZITO WA MTOTO ULIKUWA NGAPI WAKATI ANAZALIWA	Grams (4 digits) ________<br>GRAMU__________
605	Are there any vaccinations missing on the card (If yes as specifics as below)?<br><br>JE, KUNA CHANJO YOYOTE INAYOKOSEKANA KWENYE KADI (KAMA NDIYO FUATILIA HAPO CHINI)	1= Yes / NDIYO<br>2=No / HAPANA -------→ 608
606	Did your child ever receive vaccinations to prevent from getting diseases at a health facility or during campaigns?<br><br>KAMA HAPANA: JE, MTOTO WAKO ALIWAHI KUPATA CHANJO KUZUIA KUPATA MAGONJWA KWENYE KITUO CHA AFYA AU NYUMBANI?	1= Yes / NDIYO<br>2=No / HAPANA ------------------→ 608
606A	Ask for BCG (vaccination against TB that left a scar on shoulder or arm)<br><br>ULIZA KUHUSU BCG (CHANJO YA KUZUIA KUPATA UGONJWA WA KIFUA KIKUU AMBAYO ILIACHA KOVU GEGANIAU MKONONI)	1= Yes / NDIYO<br>2=No / HAPANA
606B	Pentavalent vaccine (an injection given at the same time as polio drops, how many times)<br>SINDANO INAYOCHOMWA KWENYE PAJA LA KUSHOTO WAKATI WA KUPATA MATONE YA POLIO, MARA NGAPI)?	NUMBER OF TIMES ______________ (00 if none)<br>MARA NGAPI __________________ (00 KAMA HAKUNA)
606C	Measles vaccination: an injection given against measles at children 9 months or older<br>ALIPATIWA CHANJO YA SURUA KWENYE BBEGA LA KUSHOTO?	NUMBER OF TIMES_________ (00 if none)<br>MARA NGAPI_________ (00 KAMA HAKUNA)

607	Where does your child receive the immunizations (most of the time)?<br>NI KITUO GANI CHA AFYA AMBAPO MTOTO WAKO HUWA ANAPATIWA CHANJO MARA KWA MARA?	1= Kisesa health centre<br>2= Igekemaja disp<br>3= Welamasonga disp<br>4= Isangijo disp.<br>5= Kanyama disp<br>6=Ihayabuya disp<br>10= Sekou toure<br>11= Bugando medical centre<br>12=Sumve hospital<br>13=Magu hospital<br>14= Private health facility<br>15= Other hospital or health facility
608	IF CHILD ALIVE: Did NAME sleep under bed net last night?<br>KATIKA USIKU WA KUAMKIA LEO, MTOTO ALILALA KWENYE CHANDARUA CHA KUZUIA MMBU?	1= Yes slept under bed net /ALILILALA KWNEYE NET YA KUZUIA MMBU<br>2= No did not slept under bed net /ALILILALA KWNEYE NET YA KUZUIA MMBU
609	Is this an insecticide treatment net?<br>JE HII NET INA DAWA YA KUZUIA MBU?	1 = Yes / NDIYO<br>2= No / HAPANA<br>98= Don’t know/SIJUI

SECTION 700: Corona virus epidemic knowledge and response / UFAHAMU KUHUSU UGONJWA WA CORONA		
**701**	Have you heard of COVID-19 or Corona virus?<br>**JE, UMESIKIA KUHUSU UGONJWA WA CORONA?**	1= Yes / NDIYO<br>2=No / HAPANA --------------------------------------→ END
**702**	How did you learn about it for the first time?<br><br>ULIPATAJE TAARIFA KUHUSU UGONJWA HUU WA CORONA MARA YA KWANZA?	1=Friends / RAFIKI<br>2=Radio / RADIO<br>3= TV / RUNINGA<br>4=Newspaper / GAZETINI<br>5=Health workers / MTUMISHI WA AFYA<br>6=TAZAMA personnel / MFANYAKAZI WA TAZAMA<br>7 = Social media / WhatsApp / MITANDAO YA KIJAMII<br>8 = Church / mosque / KANISANI/MSIKITINI<br>9=Through other sources / KUPITIA VYANZO VINGINE
**703**	How much do you feel you know about the COVID-19 pandemic?<br><br>UNADHANI UNAUFAHAMU UGONJWA HUU WA CORONA KWA KIASI GANI?	1=Less than I should know /CHINI YA KIWANGO KINACHOTAKIWA<br>2=A little, but not enough / KIASI LAKINI HAITOSHI<br>3=Enough / KIASI CHA KUTOSHA<br>4=A little more than most people / KIASI KIKUBWA KULIKOWATU WALIO WENGI<br>5=I am up to date on the latest research /KWA SASA HIVI MIMI NI MSHIRIKI WA TAFITI AMBAO UNAENDELEA
**704**	Do you agree with the following statements for the COVID -19?<br>JE, UNAKUBALIANO NA MAELEZO YAFUATAYO KUHUSU UGONJWA WA CORONA?	
**705**	It is possible to get COVID-19 from drinking unfiltered water<br><br>UNAWEZA KUPATA MAMBUKIZI YA CORONA KWA KUTUMIA MAJI YASIYOCHUJWA	1=Yes / NDIYO<br>2=No / HAPANA<br>98=Don't Know /SIJUI
**706**	The virus that causes COVID-19 is spread through blood<br><br>KIRUSI KINACHOSABABISHA CORONA KINASAMBAZWA KUPITIA DAMU	1=Yes / NDIYO<br>2=No / HAPANA<br>98=Don't Know / SIJUI
**707**	The virus that causes COVID-19 is spread through respiratory droplets<br><br>KIRUSI KINACHOSABABISHA CORONA KINASAMBAZWA KUPITIA MAJIMAJI YATOKANAYAO NA MFUMO WA KUPUMUA	1=Yes / NDIYO<br>2=No / HAPANA<br>98=Don't Know / SIJUI
**708**	It is possible to give the virus to someone else even if you do not show any symptoms	1=Yes / NDIYO<br>2=No / HAPANA<br>98=Don't Know /SIJUI

	JE, UNAWEZA KUMWAMBUKIZA MTU MWINGINE VIRUSI VYA CORONA HATA KAMA HUNA DALILI	
**709**	It is possible to get infected by touching a table or door handle?<br><br>JE, UNAWEZA KUAMBUKIZWA VIRUSI VYA CORONA KWA KUGUSA MEZA AU KITASA	1=Yes / NDIYO<br>2=No / HAPANA<br>98=Don't Know / SIJUI
**710**	Is it possible to get COVID-19 through unprotected sex<br>JE, UNAWEZA KUAMBUKIZWA VIRUSI VYA CORONA KWA KUfanya mapenzi bila kinga	1=Yes / NDIYO<br>2=No / HAPANA<br>98=Don't Know / SIJUI
**711**	Can a person avoid getting COVID-19 by: -<br><br>JE, MTU ANAWEZA KUEPUKA MAAMBUKIZI YA VIRUSI VYA CORONA KWA KUFANYA YAFUATYO?	
**712**	Washing hands more often?<br><br>KUNAWA MIKONO MARA KWA MARA?	1=Yes / NDIYO<br>2=No / HAPANA<br>98=Don't Know /SIJUI
**713**	Staying at home<br><br>KUBAKI NYUMBANI	1=Yes / NDIYO<br>2=No /HAPANA<br>98=Don't Know /SIJUI
**714**	Wearing a mask<br><br>KUVAA BARAKOA	1=Yes/ NDIYO<br>2=No / HAPANA<br>98=Don't Know/ SIJUI
**715**	Going to church<br><br>KWENDA KANISANI	1=Yes / NDIYO<br>2=No / HAPANA<br>98=Don't Know/ SIJUI
**716**	Maintaining distance from other people<br><br>KUKAA MBALI NA WATU WENGINE	1=Yes / NDIYO<br>2=No /HAPANA<br>98=Don't Know/ SIJUI
**717**	No travel to Mwanza City<br><br>KUTOKWENDA MWANZA MJINI	1=Yes/ NDIYO<br>2=No / HAPANA<br>98=Don't Know /SIJUI
**718**	Not having unprotected sex<br><br>KUTOKUFANYA MAPENZI BILA KINGA	1=Yes / NDIYO<br>2= No / HAPANA<br>98= Don't Know/ SIJUI
**719**	Avoid markets and crowds<br><br>EPUKA KWENDA SOKONI NA MIKUSANYIKO	1=Yes/ NDIYO<br>2=No / HAPANA<br>98=Don't Know / SIJUI
**720**	Avoid hand shakes<br><br>EPUKA KUSHIKANA MIKONO	1=Yes / NDIYO<br>2=No / HAPANA<br>98=Don't Know / SIJUI
**721**	Do you do anything to avoid getting COVID-19?<br><br>JE, WEWE BINAFSI UNAFANYA CHOCHOTE KUEPUKA KUAMBUKIZWA VIRUSI VYA CORONA?	1=Yes / NDIYO<br>2=No / HAPANA<br>98=Don't know/SIJUI

**722**	What are you doing to avoid infection by COVID-19 virus?<br><br>UNAFANYA NINI KUEPUKA KUPATA MAAMBUKIZI YA VIRUSI VYA CORONA?<br><br>ZUNGUSHIA YOTE ATAKAYOTAJA	1=Avoid hand shakes/ SISHIKANI MIKONO NA WATU<br>2=Avoid markets and crowds/ NAEPUKA KWENDA SOKONI NA MIKUSANYIKO<br>3=Not having unprotected sex/ SIFANYI MAPENZI BILA KINGA<br>4=No travel to Mwanza City / SIENDI MWANZA MJINI<br>5=Maintaining distance from other people / NINAKAA MBALI NA WATU WENGINE<br>6=Washing hands more often? / NINI NAWA MIKONO MARA KWA MARA?<br>7=Staying at home /NINABAKI NYUMBANI<br>8= Others (mention)/ NYINGINE (TAJA) _____________
**723**	Do you think that there is a chance that you will become infected with COVID-19 in the future?<br><br>JE, UNADHANI KUNA UWEZEKANO WEWE UKAPATA MAAMBUKIZI YA VIRUSI VYA CORONA?	1=No chance at all /HAIWEZEKANI KABISA --→725<br>2= Small chance / UWEZEKANO MDOGO<br>3=Likely / INAWEZEKANA<br>4=Almost certain / NITAAMBUKIZWA<br>98=Don't know / SIJUI -→725
**724**	why she thinks so?<br>KAMA JIBU LA 823 SIYO 1 AU 5. ULIZA KWANINI ANAFIKIRIA HIVYO?	1=Do hand shakes / NASHIKANA MIKONO NA WATU<br>2=Don't avoid markets and crowds / SIE PUKI KWENDA SOKONI NA MIKUSANYIKO<br>3=I have unprotected sex / NAFAFANYI MAPENZI BILA KINGA<br>4= I do go to Mwanza City / NASAFIRI KWENDA MWANZA MJINI<br>5= Don't maintaining distance from other people / SIKAI MBALI NA WATU WENGINE<br>6=Don't do hand wash more often? / SINAWI MIKONO MARA KWA MARA?<br>7=Don't stay at home / SIBAKI NYUMBANI<br>8= Others (mention)/ NYINGINE (TAJA) _____________
**725**	If you were to become infected, how severe do you think COVID-19 would be for you?<br>KAMA IKITOKEA UKAAMBUKIZWA, UNADHANI UTAUGUA SANA AU KIASI, KIDOGO TU AU HUTAPATA DALILI ZOZOTE?	1=Very severe / NTAUGUA SANA<br>2=Severe / NTAUGUA<br>3=Mild severe /NITAUGUA KIASI<br>4=No symptoms / SITAKUWA NA DALILI ZOZOTE<br>98= Don't know / SIJUI
**725**	Why do you say so?<br>KWANINI UNASEMA HIVYO?	1=Got a strong body / MWILI UNA NGUVU<br>2=I usually don't get illnesses /SINA KAWAIDA YA KUUGUA<br>3=I am protected as I always use traditional medications / NINA KINGA KWANI NATUMIA MITISHAMBA MARA KWA MARA<br>4 = I am already not so healthy/SINA AFYA NJEMA
		5= other responses/MAJIBU MENGINE

Thank you for taking time to talk to me!!
